# Parallels between DNA and collagen – comparing elastic models of the double and triple helix

**DOI:** 10.1038/s41598-017-12878-3

**Published:** 2017-10-16

**Authors:** Fei Xu, Hongning Zheng, Nicolas Clauvelin, Xiang-Jun Lu, Wilma K. Olson, Vikas Nanda

**Affiliations:** 1 School of Biotechnology, Jiangnan University, 1800 Lihu Ave., Wuxi, Jiangsu 214122 China; 2 Department of Chemistry and Chemical Biology, Rutgers University, 610 Taylor Road, Piscataway, NJ 08854 USA; 3 Department of Biological Sciences, Columbia University, New York, NY10027 USA; 4 Department of Biochemistry and Molecular Biology and the Center for Advanced Biotechnology and Medicine, Robert Wood Johnson Medical School, Rutgers University, 679 Hoes Lane West, Piscataway, NJ 08854 USA

## Abstract

Multi-stranded helices are widespread in nature. The interplay of polymeric properties with biological function is seldom discussed. This study probes analogies between structural and mechanical properties of collagen and DNA. We modeled collagen with Eulerian rotational and translational parameters of adjacent rungs in the triple-helix ladder and developed statistical potentials by extracting the dispersion of the parameters from a database of atomic-resolution structures. The resulting elastic model provides a common quantitative way to describe collagen deformations upon interacting with integrins or matrix metalloproteinase and DNA deformations upon protein binding. On a larger scale, deformations in Type I collagen vary with a periodicity consistent with the D-periodic banding of higher-order fibers assemblies. This indicates that morphologies of natural higher-order collagen packing might be rooted in the characteristic deformation patterns.

## Introduction

Biomacromolecules often adopt multi-stranded helical structures, such as the DNA double helix and the collagen triple helix. Regardless of the differences in biological function and chemical make-up, the common molecular organization of these systems — regular, recurring arrangements of identical polymer backbones stabilized by inter-strand associations — hint of common strategies that the structures might invoke in response to changes in the local environment or upon formation of higher-order assemblies. Inspired by algorithms previously used to characterize DNA, we have developed a statistical mechanical approach to describe collagen structure and mechanical behavior. The DNA double helix is well characterized in terms of its sequence-specific deformability and the relationship of these local features to its overall polymeric behavior^1,2^. The degrees of freedom between two adjacent base pairs in DNA are reduced to three translational and three rotational parameters^3,4^. Variation in these parameters observed across a dataset of high-resolution nucleic acid structures can be transformed into a set of empirical elastic functions that describe DNA deformability. This deformability underlies mechanical aspects of processes such as protein-induced DNA looping, DNA cyclization, and genomic nucleosome positioning that require effective modeling of hundreds of bases^5–8^. As with DNA, the biological function of collagen requires deep understanding of structure and energetics at both the local scale in terms of binding to extracellular matrix proteins, and at a larger scale for collagen fiber assembly and mechanics^9,10^.

Parametric models of collagen structure generally treat the three-stranded structure as a rigid rod, presumably due to the preponderance of short peptide structures in the Protein Data Bank that exhibit little curvature. The sequences of each chain contain repeated (Gly-X-Y) triplets, where X is frequently proline and Y is the post-translationally modified proline derivative, 4R-hydroxyproline. Individual chains adopt an extended, left-handed poly-proline type II (PPII) local helix, which supercoils upon formation of the triple-helix. In existing parametric models, each PPII strand is represented in terms of twist and rise along the principal cylindrical axis that relate residues in successive turns of the helical chain^11^. A more accurate representation of the Gly position, which lies closest to the common helical axis, is obtained if a cylindrical coordinate system with eleven parameters is used to describe each PPII chain^12^. Similar accuracy can be achieved describing three chains by one set of parameters by dividing the triple-helix into Gly/X/Y layers made up of residues from the different chains^13^.

A key limitation of existing models of collagen is that they do not describe bending and shearing degrees of freedom in the triple helix. Even in short peptides, such deviations from a straight rod are observed upon binding matrix proteins such as integrin and matrix metalloproteinase^14,15^. Some collagen sequences are intrinsically curved in the absence of bound proteins^16,17^. The elastic model developed in this study adapts the El Hassan-Calladine description of nucleic acid base-pair step parameters^18^ to characterize the Gly/X/Y layers in the triple helix, appropriately modeling bending and shearing and eliminating the need for a rigid-rod approximation.

In addition to providing insight into peptide models of collagen, the parameterization can be used to examine the *in situ* structure of type I collagen microfibrils, the major form of natural collagen, obtained by X-ray fiber diffraction^19^. The type I collagen microfibril is a supermolecular assembly composed of five long triple helices (~300 nm long), among which two neighboring triple helices are staggered ~67 nm with respect to each other, forming a D-periodic banding pattern seen with electronic microscopy^20^. Various correlations between triple helix conformations, ligand binding sites, and fibrillar packing have been noted^21,22^.

The deformability of collagen is central to its function from ligand binding to fibrillogenesis^9,10^. Effectively modeling these processes requires both sufficient resolution to incorporate sequence contributions to deformation, and sufficient scalability to calculate energetics on large systems of thousands of residues or more. Existing models are either coarse-grained, grouping multiple residues into beads with limited sequence information included^23^, or all-atom simulations^24–26^ providing detailed chemical information, but limited in temporal and spatial scope by the cost of large detailed calculations. The elastic model offered here bridges aspects of coarse-grained and atomistic treatments; it may be applied to large systems with relatively little computational cost while considering key molecular features at the local sequence level.

A number of intriguing parallels between DNA and collagen are made evident by the elastic model. Deformations of both polymers can be induced by either direct ligand binding or indirect effects from multiple-domain cooperation of bound ligands. Two case studies – integrin binding and matrix metalloproteinase binding — are examined in detail and presented next to counterparts in protein-DNA interactions. At a larger length scale, global patterns of deformation in the structure model of Type I collagen are consistent with the periodicity of packing of individual triple helices within the microfibril.

## Methods

### Cα-triangle reference frame

**Figure 1:**
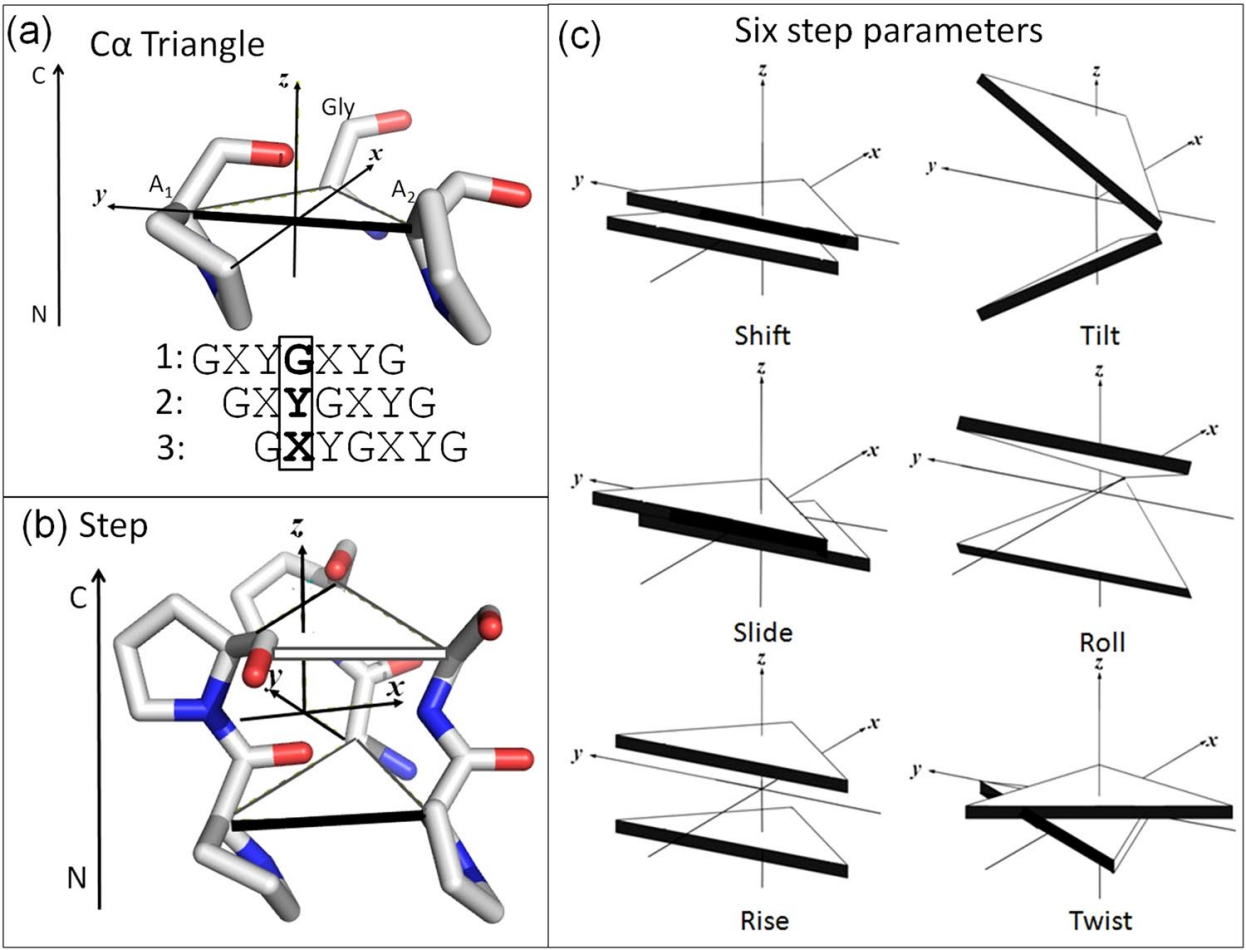
Defining geometric parameters of a collagen triple helical step. (**a**) The reference frame of a Cα triangle (see Methods for details). (**b**) The middle reference frame between successive triangles of a triple helical step. (**c**) Illustration of positive values of the six step parameters. Triangles are obtained with procedures in the 3DNA software package^3^.

Collagen is treated as a series of triangles whose vertices are the Cα atoms from a Gly in one strand and the Cα atoms from the nearest non-Gly residues (A1 and A2) in the two other strands (Fig. 1(a)). A standard reference frame is built upon each Cα-triangle, determined in this example by the fourth, third and second residues in the first, second and third chains from the structure of [GPPGPPG]_3_ (PDB ID: 1ITT)^27^. The *y*-axis is defined by the vector connecting Cα atoms A2 to A1. The origin is taken as the midpoint between Cα atoms A1 and A2. The *x*-axis is perpendicular to the *y*-axis and forms an acute angle with the vector connecting the origin to the Gly Cα. The *z*-axis is the cross product of the *x-*and *y-*axes with positive values in the direction of the global axis from the N- to C-termini of the triple helix. The reference frame of a given Cα-triangle is obtained by superimposing the standard frame onto the given one with a least-squares fitting procedure^28^.

### Triple-helical step parameters

Two successive Cα triangles form a triple-helical step (Fig. 1(b)), in which the relative position and orientation of the two Cα triangles are described with six geometric, or step, parameters. The three translational parameters along the *x*-, *y*-, and *z*-axes are termed Shift, Slide, and Rise, respectively, while the three rotational ones are called Tilt, Roll, and Twist (Fig. 1(c)). The step parameters are computed with respect to a middle reference frame using the numerical implementation of El Hassan-Calladine^18^ within the 3DNA suite of programs^3^.

### Knowledge-based elastic function

A knowledge-based elastic function is set up to estimate the cost of collagen deformations. The deformation score, *E*, of a triple-helical step is taken as the sum of the pair-wise elastic contributions over the six step parameters.

1 $$E={E}_{0}+\frac{1}{2}\sum _{i=1}^{6}\sum _{j=1}^{6}{f}_{ij}{\rm{\Delta }}{\theta }_{i}{\rm{\Delta }}{\theta }_{j},$$

where *E*_0_ is the baseline, assumed as zero here, and ${\rm{\Delta }}{\theta }_{i}={\theta }_{i}-\,\overline{{\theta }_{i}}$ is the deviation of the *i* ^th^ step parameter from its ‘equilibrium’ state, $\overline{{\theta }_{i}}$. The equilibrium states and force constants, *f*_*ij*_, are derived from a step-parameter dataset extracted from 40 well-resolved collagen structures including 11 collagen-protein complexes (Table S1). The step parameter identities $i,j=1\cdots 6$ correspond respectively to Shift, Slide, Rise, Tilt, Roll and Twist. Extreme values are eliminated with a culling procedure to avoid bias effects and enhance data clustering (see Method in Supplementary Material, Fig. S2). The reported values of $\overline{{\theta }_{i}}$ are the mean values over the culled dataset. The parameter dispersion is analyzed with an inverse harmonic analysis to obtain the elastic force constants^1^. The covariance of the culled step parameters is collected in the covariance matrix *C* with elements ${c}_{ij}={s}_{i}{s}_{j}-{s}_{i}{s}_{j}$, where *i* and *j* are the step parameter identities, and the force constant matrix *F* with elements *f*_*ij*_ is obtained by taking the inverse of *C*.

## Results and Discussion

### Similar shapes of Cα triangles

DNA double helices can be effectively represented as a series of planes. Similarly, collagen triple helices can be divided into triangles. More than 900 Cα triangles were identified in forty triple-helical peptide structures (Table S1). The dimensions were uniform and close to an equilateral triangle with side lengths of ~5 Å (Fig. S1), allowing a facile representation of the triple-helix as a series of triangles. We adopt the nomenclature of Bella^13^ for describing the sequence content of Cα triangles, dividing them into three groups: GP_2_, GP_1_ and GP_0_, with the subscript representing the number of imino (Pro/Hyp) groups in the Gly/X/Y triads. Due to the high stability of Gly-Pro-Hyp triplets^29^, the number of GP_2_ triangles (754) is far greater than the number of GP_1_ (135) and GP_0_ (64) triangles. The side lengths of the GP_2_ and GP_1_ triangles are slightly shorter than those of the GP_0_ triangles, indicating tighter packing of these layers.

### Sequence dependence of step parameters

The six step parameters define the relative geometry between adjacent triangles in the triple helix. In the case of DNA, sequence content affects the mean values of the step parameters. The same is noted for collagen where the proline content of the two neighboring triangles systematically affects the mean values of parameters (Table 1). As all the triple helical structures are homotrimers, the numbers of Pro/Hyp residues in neighboring triangles differ by no more than one. When the imino content decreases from GP_2_-GP_2_ to GP_0_-GP_0_, there are decreases in |Tilt| and |Roll|, and increases in |Shift| and |Twist|. Based on the value similarities, the seven groups can be approximated by three groups, i.e., high-imino (GP_2_-GP_2_), mid-imino (GP_x_-GP_y_, x < 2 or y < 2), and low-imino (GP_0_-GP_0_) groups.

**Table 1:** Mean and root-mean-square deviations of triple helical step parameters extracted from 40 well-resolved collagen X-ray crystal structures.

Step	Step	Shift	Slide	Rise	Tilt	Roll	Twist
	number	(Å)	(Å)	(Å)	(°)	(°)	(°)
GP_2_-GP_2_^*^	642	−4.24 ± 0.18	0.84 ± 0.17	3.25 ± 0.07	−11.14 ± 1.51	9.46 ± 1.94	−101.72 ± 2.84
GP_2_-GP_1_	31	−4.32 ± 0.14	0.85 ± 0.22	3.22 ± 0.06	−10.28 ± 1.53	8.52 ± 2.5	−103.91 ± 3.02
GP_1_-GP_2_	29	−4.35 ± 0.14	0.79 ± 0.21	3.23 ± 0.08	−10.38 ± 1.41	8.39 ± 1.47	−103.47 ± 2.79
GP_1_-GP_1_	77	−4.41 ± 0.21	0.78 ± 0.18	3.20 ± 0.10	−9.59 ± 1.61	7.56 ± 2.02	−104.38 ± 2.83
GP_1_-GP_0_	22	−4.45 ± 0.24	0.75 ± 0.14	3.20 ± 0.09	−9.07 ± 2.04	6.69 ± 2.24	−105.19 ± 4.27
GP_0_-GP_1_	21	−4.48 ± 0.22	0.65 ± 0.22	3.21 ± 0.09	−9.25 ± 2.66	6.67 ± 2.20	−104.99 ± 3.74
GP_0_-GP_0_	42	−4.56 ± 0.24	0.75 ± 0.21	3.14 ± 0.15	−7.89 ± 2.63	5.97 ± 2.25	−107.48 ± 3.74

*Subscripts are the numbers of imino (Pro/Hyp) residues in a Gly/X/Y triad.

The steps are divided into 7 groups according to the number of Pro or Hyp residues. GP_2_-GP_0_ and GP_0_-GP_2_ steps were not observed due to the preponderance of homotrimers in the database.

**Figure 2:**
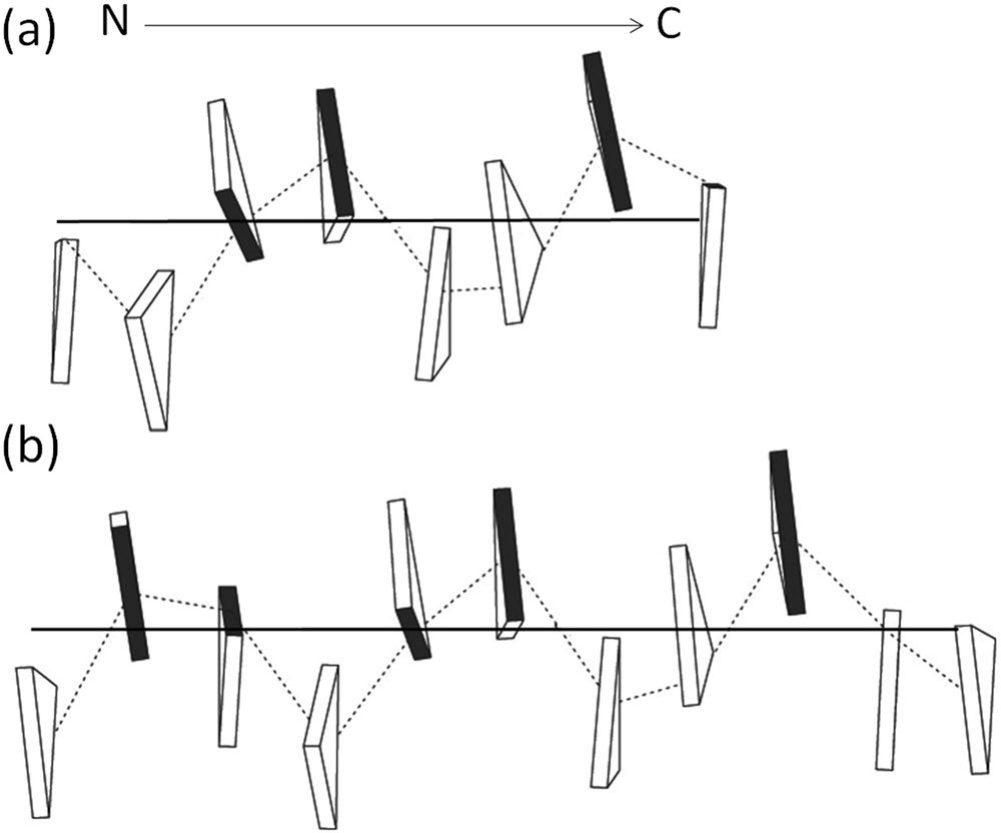
Stacking models of (**a**) GP_2_-GP_2_, and (**b**) GP_0_-GP_0_ steps built from their mean step values, where the subscripts are the numbers of imino (Pro/Hyp) residues in a Gly/X/Y triad. The edges of the triangles connecting the two Cα atoms of non-Gly residues are shaded in black.

The super-helical repeat, or pitch, of collagen ranges between a more loosely wound 10 residues per 3 turns helix in the low-imino sequence GPRGNRGERGSE, and a tightly wound 7 residues per 2 turns helix in the high-imino sequence (POG)_9_^27,30^. According to Bella’s parameterization based on twist, the pitch has a strong sequence dependence, with high proline content favoring the tightly wound state^13^. The same sequence dependence is recapitulated with the step parameters. The mean values of the step parameters averaged over the GP_2_-GP_2_ and GP_0_-GP_0_ steps were used to build sequence-dependent models using a reversal of the procedure for determining the step parameters. The GP_2_-GP_2_ model approximates a 7/2 helix and the GP_0_-GP_0_ model approximates a 10/3 helix (Fig. 2). The calculation of super helical periodicity, with the algorithms of Miyazawa^31^ (see Method in Supplementary Material), confirms the imino-dependent periodicity observed in the stacking models. The GP_2_-GP_2_ helical repeat is 3.49 steps/turn, while that of GP_0_-GP_0_ is 3.36 steps/turn (Table S2). The repetition of GP_x_-GP_y_ steps (x < 2 or y < 2) yields non-canonical structures intermediate between 7/2 and 10/3 helices with 3.43 steps/turn (~17 steps/5 turns). The imino-dependent helical periodicities parallel sequence-dependent helical structures of DNA, where A/T-rich DNA sequences tend to form *B* DNA and G/C sequences *A* DNA^32,33^.

### Sequence-dependent deformability

In addition to using mean values of step parameters to connect sequence and structure, the variance provides information on elastic force constants that describe deformability of the triple helix. Higher imino content correlates with higher conformational rigidity. The matrix containing the pair-wise covariance of step parameters describes an imaginary six-dimensional space. The spatial volume, obtained from the product of the matrix eigenvalues, reflects the conformational rigidity. The order of these values is GP_2_-GP_2_ (0.0009) < GP_x_-GP_y(x<2, y<2)_ (0.0021) < GP_0_-GP_0_ (0.0049).

**Figure 3:**
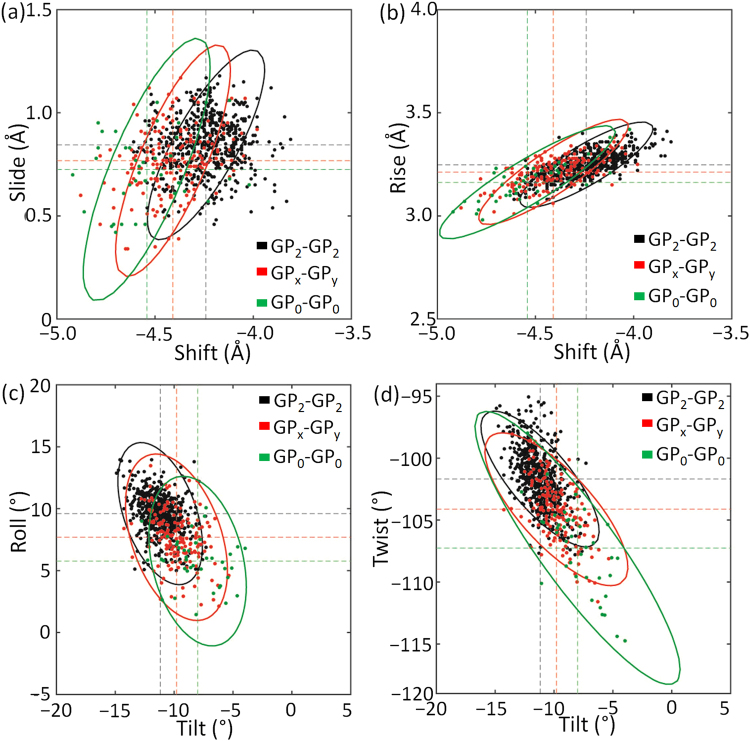
Derived energy contours of (**a**) Slide-Shift, (**b**) Rise-Shift, (**c**) Roll-Tilt, and (**d**) Twist-Tilt with high (GP_2_-GP_2_), middle (GP_x_-GP_y_, x < 2 or y < 2), and low (GP_0_-GP_0_) imino contents. The level of the elliptical equi-potential contours corresponds to three times the root-mean square deviation (3*σ*) of the data points.

Inversion of the covariance matrix gives the pair-wise elastic force constants (Table S3(a)). The extent of conformational coupling between step parameters and their dependence on sequence can be visualized with elliptical 2D projections of the six-dimensional covariance space (Fig. 3). Here if the inclination of an ellipse is 45 ± 25° and the long/short axis ratio is greater than 1.2, the corresponding parameters are defined as highly coupled. The values of Slide-Shift, Slide-Rise and Roll-Twist are correlated over the three imino groups (Table S3(b)). Notably, the Roll and Tilt of GP_0_-GP_0_ steps are not found to be highly coupled, with an inclination angle larger than 70°. Over the three imino groups, the narrow Rise-Shift ellipses in the Rise dimension reflect the restricted stretching or compressing movements along the *z*-axis. The significant variation of the Twist-Tilt ellipse area results from the wide dispersion of Twist in GP_0_-GP_0_ steps.

The linear correlations are consistent with the couplings derived from the covariance space. The 0.001 significance level chosen here is identical to that used in a similar conformational study of DNA double helices^34^. Rise-Shift, Twist-Tilt, Shift-Twist and Rise-Twist, show strong correlations over the three imino-content groups (Table S4).

### Direct deformation upon integrin binding

Cell adhesion and migration processes are meditated through integrin proteins that bind to specific sites on matrix proteins. The α2β1 integrin recognizes a GFOGER sequence on collagen^14^. Three states, represented by the integrin-free GFOGER structure and by 1:1 and 2:1 integrin-collagen complexes (PDB ID: 1Q7D, 1DZI, 4BJ3)^14,35,36^, were analyzed using the methods and potentials described above. These structures were not included in the database used to derive the elastic model. Although the resolutions of these structures vary from 1.8 to 3.04 Å, the elastic models are robust to uncertainty in coordinates stemming from lower resolution. The deformation scores remain similar when atomic coordinates in the X-ray structures are perturbed numerically (Fig. S3).

**Figure 4:**
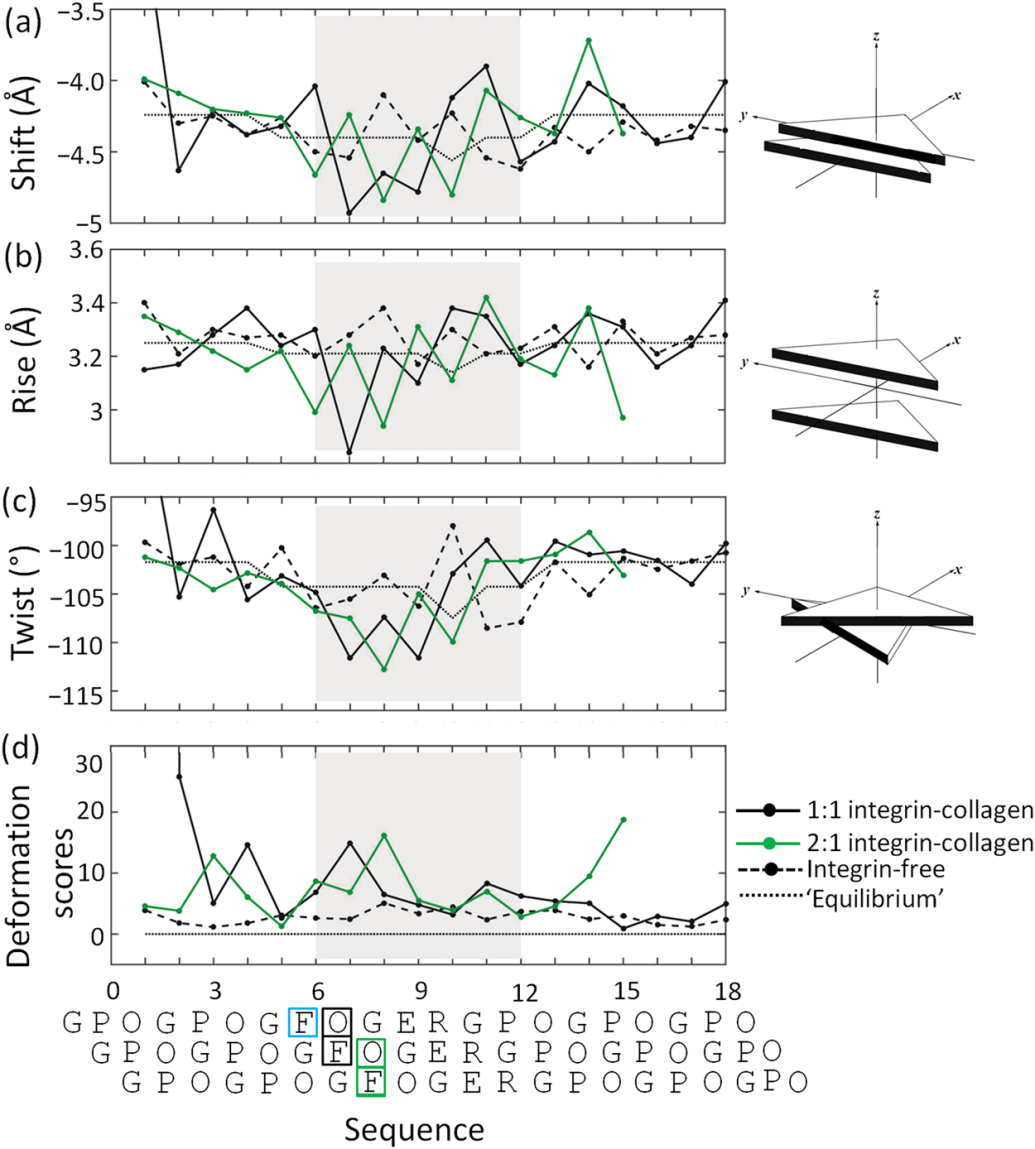
(**a**) Shift, (**b**) Rise, (**c**) Twist, (**d**) deformation scores of integrin-bound complexes (PDB: 1DZI, 4BJ3) and integrin-free (DPB: 1Q7D) triple helices with the same sequence^14,35,36^. The mean step parameter values, or ‘equilibrium states’ are listed in Table S3. The integrin-bound region is shaded in grey. The sequences are aligned by step number. The Phe and Hyp residues contacted by integrin Asn154 and Leu296 are framed by color-coded rectangles: black for the 1:1 complex; green for integrin molecule A; light blue for integrin molecule B (contact only to Phe).

The marginal deformation scores (eq. 1) of integrin-free collagen are near equilibrium values. Some deviations from mean step parameters are observed in the unbound state including increased Rise and decreased |Shift| in Step 8 (GOF-EGO), and increased |Tilt| and decreased |Twist| in Step 10 (REG-GRE) (Figs 4 and S4). However, these deviations compensate each other such that the deformation score of each step is lower compared to integrin-bound states.

**Figure 5:**
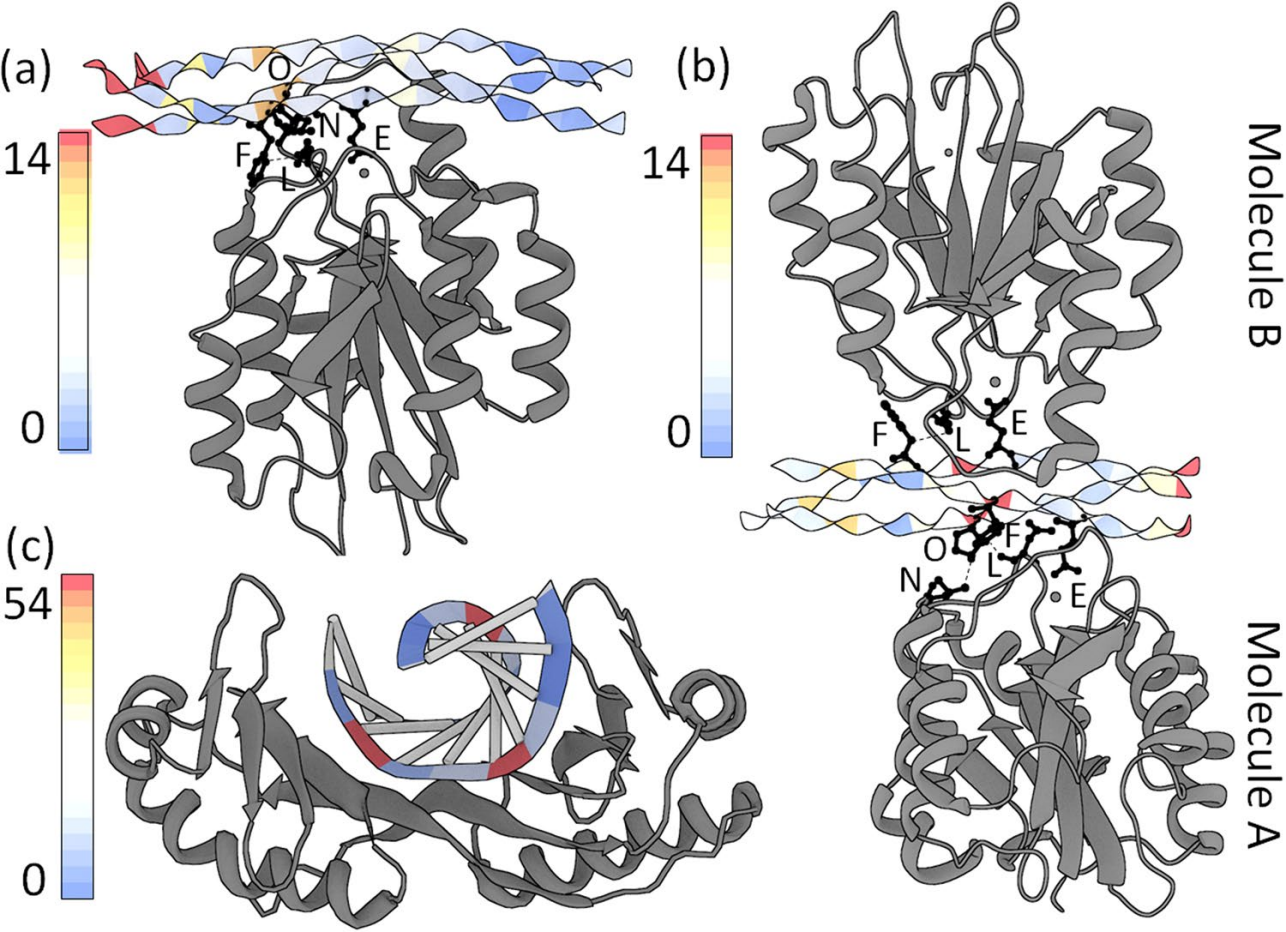
Deformed triple helices in integrin-collagen complexes and double helices in a protein-DNA complex. The deformation scores are color-coded and mapped on ribbon representations of collagen in the (**a**) 1:1 and (**b**) 2:1 integrin-collagen complexes (PDB ID: 1DZI, 4BJ3)^14,36^. The same integrin residues, Asn154 and Leu296, contact Phe and Hyp in the various chains of the triple helices. Contacting pairs are shown in ball-and-sticks. The Glu residues involved in the three metal ion-dependent adhesion sites (MIDAS) are also shown in ball-and-stick representations. (**c**) Corresponding deformation scores and images of the TATA-box DNA-protein complex (PDB ID: 1YTB)^37^. See supplement for calculation of DNA deformation scores.

The 2:1 complex is observed if a point mutation, E317W, is introduced. Although this mutation is located more than 20 Å away from the binding site, it induces the simultaneous binding of two integrin ligands (molecules *A* and *B*) via presumed allosteric interactions. In the 1:1 complex, the leading chain Hyp and middle chain Phe at the first Cα triangle of Step 7 (OFG-GOF) come in close contact (≤4.0 Å) with integrin Asn154 and Leu296 (Fig. 5) resulting in a high deformation score. In the 2:1 complex, the same Asn154 and Leu296 residues of molecule *A* make close contacts to the Phe and Hyp in the first Cα triangle of Step 8 (GOF-EGO). The highly deformed step thus shifts from Step 7 in the 1:1 complex to Step 8 in the 2:1 complex. The contacted residues, Phe and Hyp, are respectively shifted from the leading and middle chains to the middle and trailing chains. The step parameters contributing to the high deformation scores are similar, arising in both Step 7 in the 1:1 complex and Step 8 in the 2:1 complex from increased |Shift| and decreased Rise (Fig. 4). An increase in |Tilt| also makes a large contribution to the high deformation score of Step 8 in the 2:1 complex. Following a similar pattern, Asn154 of integrin molecule *B* in the 2:1 complex makes a close contact with the Phe in the leading collagen chain, causing a moderately high deformation score at Step 6 (FGO-OFG). The deformation at Step 6 is also caused by increased |Shift| and decreased Rise. This suggests that the Phe-Asn and Hyp-Leu interaction pairs play an essential role in the integrin recognition of the GFOGER motif.

Compared to the highly distorted step containing Phe, the Glu-containing steps have quite low deformation scores in both the 1:1 and 2:1 complexes. Without any disruption of triple helix conformations, the Glu side chains are well extended and inserted into the C-terminal loop of the β strand forming the metal ion-dependent adhesion site (MIDAS)^14^. Except for the deformation at GFOGER, the N-terminus hosting GPO triplets is also distorted, which might be due to lattice packing.

The total bending component, $\sqrt{{{\rm{Tilt}}}^{2}+{{\rm{Roll}}}^{2}}$, offers a quantitative measure of local collagen bending. The bending components of the integrin-free collagen are much closer to the ‘equilibrium’ states with smaller values of oscillation compared to the 1:1 and 2:1 complexes (Fig. S4). The degrees of bending are similar in the 1:1 and 2:1 integrin complexes except for the increased values of |Tilt| and Roll at Step 11 of the 1:1 complex. The bending at multiple sites could be cumulative or cancel one another, depending upon the relative orientation between the sites. The bending components of the 1:1 and 1:2 complexes, however, primarily differ at step 11 (17.5° in 1:1 vs. 12.6° in 2:1 complexes), with a 4.9° difference consistent with the independently measured ~5° bending in the 1:1 complex^35^.

In the case of integrin binding, collagen deformation is localized at the site of binding. Like this example, the highly bent DNA in the TATA-box DNA-protein complex, a classic example of protein-induced DNA deformation with significantly increased Roll and decreased Twist, is also localized (Fig. 5(c)) (PDB ID: 1YTB)^37^. The TATA-box DNA-protein complex shows an extremely large degree of DNA bending even among protein-DNA complexes, which is not comparable to the modest bending components of collagen. Although the deformations of collagen and DNA are expressed in terms of different parameters owing to their different chemical features and backbone conformations, the distortions in both molecules could be induced by direct contacts from ligands.

### Indirect deformation upon MMP1 binding

**Figure 6:**
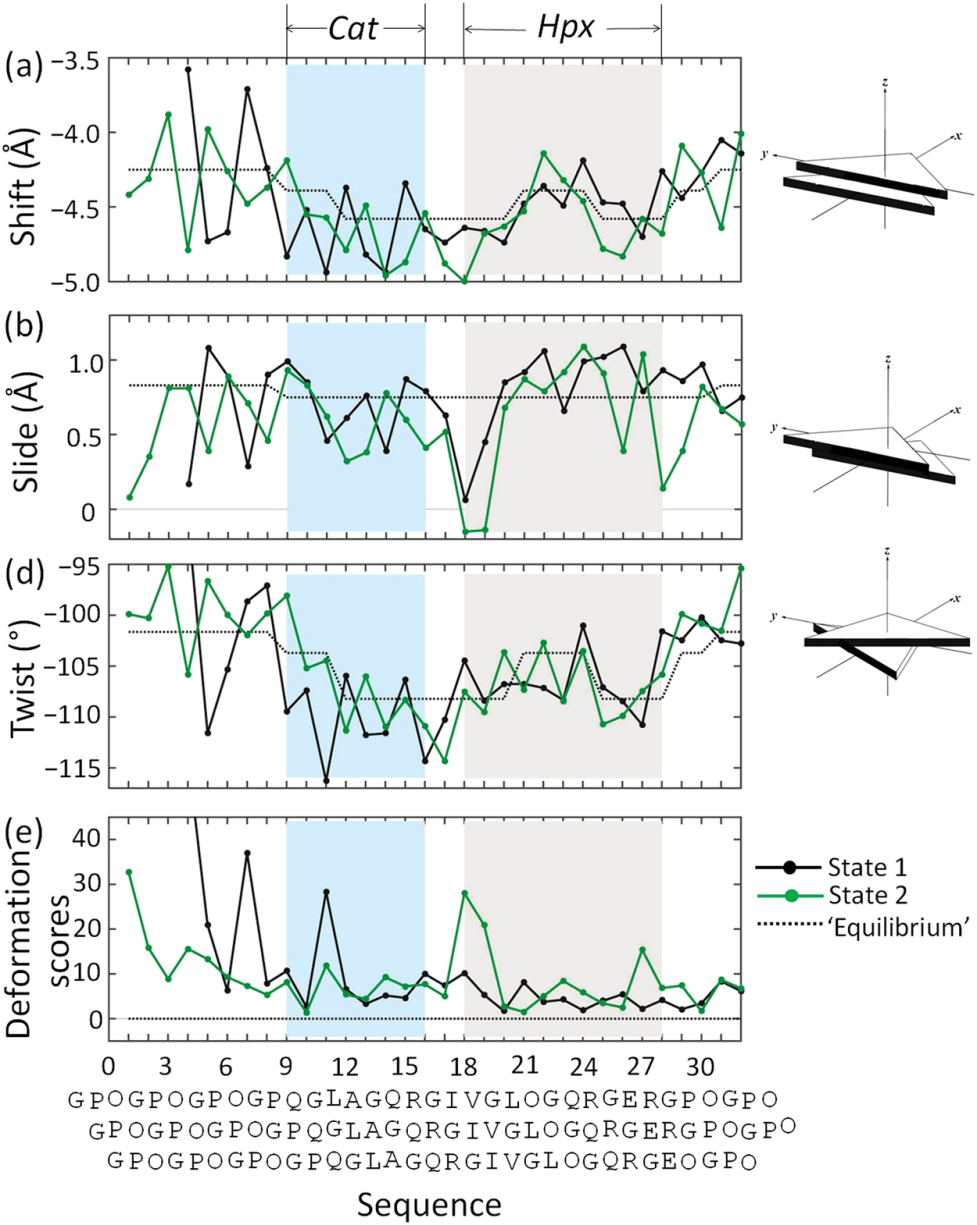
(**a**) Shift, (**b**) Slide, (**c**) Twist, (**e**) deformation scores of two states of matrix metalloproteinase 1(MMP1)-bound triple helices (PDB: 4AUO)^15^. The mean step parameter values, or ‘equilibrium states’, are listed in Table S3. The sequences contacted (atom-atom distance ≤ 4 Å) by MMP1 N-terminal catalytic (*Cat*) and C-terminal hemopexin (*Hpx*) domains are shaded in light cyan and grey, respectively. The sequences are aligned along the step number.

**Figure 7:**
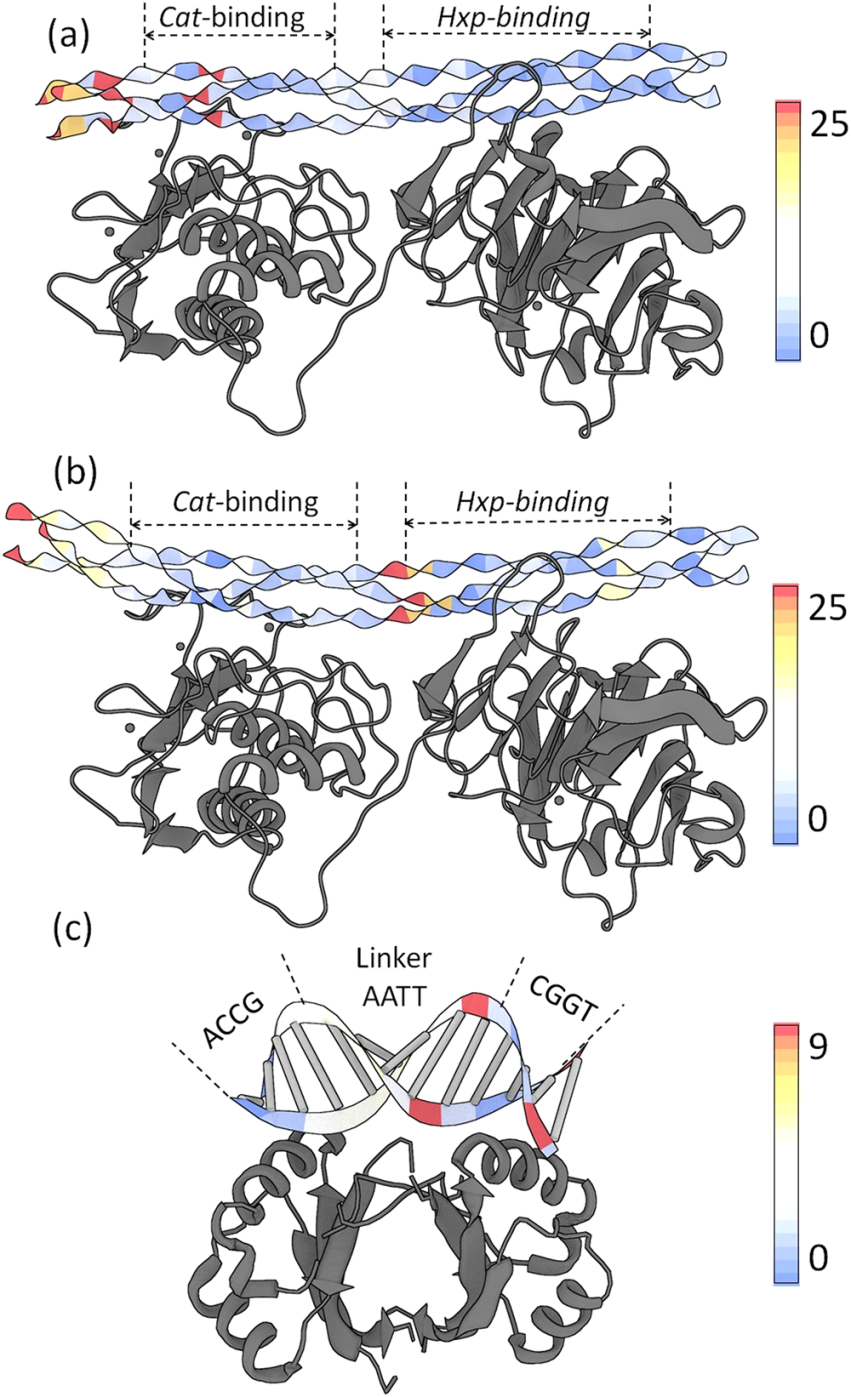
Collagen and DNA deformations induced by the indirect contacts of ligands. The deformation scores are color-coded and mapped on two states of the triple helices, (**a**) and (**b**), in the MMP-collagen complexes (PDB ID: 4AUO)^15^, and (**c**) the HPV E2 protein-DNA complex (PDB ID: 1JJ4)^42^.

The high-resolution structure of matrix metalloproteinase 1 (MMP1) bound to a collagen mimetic peptide has two distinct conformations of the complex in the asymmetric unit (PDB-ID: 4AUO)^15^. Analyses of the step parameters and deformation scores of these two states reveal collagen deformations induced by both direct contacts and indirect readout from the catalytic (*Cat*) and hemopexin (*Hxp*) domains of MMP1. In State 1, the deformation of Step 11 (GQP-LGQ) is induced by close contacts from the MMP1 *Cat* domain. The high deformation score mainly reflects increased values of |Shift| and |Twist| (Figs 6 & 7). In contrast, the mostly strongly deformed region in State 2 occurs at Step 18 (IGR-VIG), located between the sites where the *Cat* and *Hxp* domains contact collagen. Only the Val and Ile at the second triangle of the step make close contacts with the *Hxp* domain. Notably, these direct/indirect contacts are defined in terms of the distances between different molecules, such as biopolymers and their ligands, and not within the same molecule^38,39^. Usually the cutoff values of close contacts are defined in the range from 3.2 to 4.0 Å^40^. When a more restricted cutoff, *i.e*. 3.2 Å, is used, the second triangle (VIG) of step 18 could be defined as an indirect contact by the *Hxp* domain. However, a 4.0 Å cutoff is used here in order to be consistent with the cutoff used in the original crystal structure^15^. The deformation at Step 18 entails increased |Shift| and decreased Slide. Although none of the six residues in Step 17 comes in close contact with either the *Cat* or the *Hxp* domain, Step 17 is also deformed with the increased values of |Shift| and |Twist|. The coupling between the Shift and Twist gives rise to the low total deformation score of Step 17, exhibiting how conformational distortion can be accommodated. The different modes of collagen deformation, in States1 and 2, suggest the important roles of the deformability at the linker region between the *Cat* and *Hxp* domains. Consistently, the replacement of the amino triplet at the linker region with GPO causes a significant decrease in the hydrolysis efficiency of MMP1^41^.

Linker region deformability has also been observed in DNA bound by multi-domain proteins. For example, highly flexible AT-rich linker sequences enhance the binding affinity of the papillomavirus (HPV) E2 protein to ACCG-*nnnn-*CGGT sequences. When the linkers are TAAT or ATAT, the binding affinity increases up to ~3000 fold over that of rigid GC-rich sequences, such as CGCG or GGCC^2,42^. The linker region bends at the AT:AT step with a large negative Roll. A large Tilt value leads to a high deformation score at the TC:GA step located between the linker and the CGGT sequences. The distorted conformations of both collagen and DNA at linker regions (Fig. 7(c)) hint of similar mechanisms in which the intrinsic deformability along a thin biopolymer may facilitate recognition by multi-domain protein ligands.

### Collagen microfibril packing

The elastic model provides insights into how collagen deformation plays a role in fibrillar assemblies. 1008 steps were identified in an atomic model constructed by Varma *et al*.^25^ from a 5.16 Å-resolution *in situ* fiber diffraction structure of Type I collagen^19^. The mean values of the step parameters in the natural collagen are similar to those of the crystal structures but with much larger deviations (Table S6 vs. Table 1), which may be due to the differences in structural resolution of the peptide and natural collagen structures. The elastic force constants are derived from the highly clustered dataset of well-folded triple helices in X-ray crystal structures. The cost of deformation from these closely related states are thus large. When the force constants are applied to the *in situ* model of natural collagen, the deformation scores of natural collagen (mean 411.6 ± 613.8) are significantly higher with much larger variations compared to those in the crystal structures.

**Figure 8:**
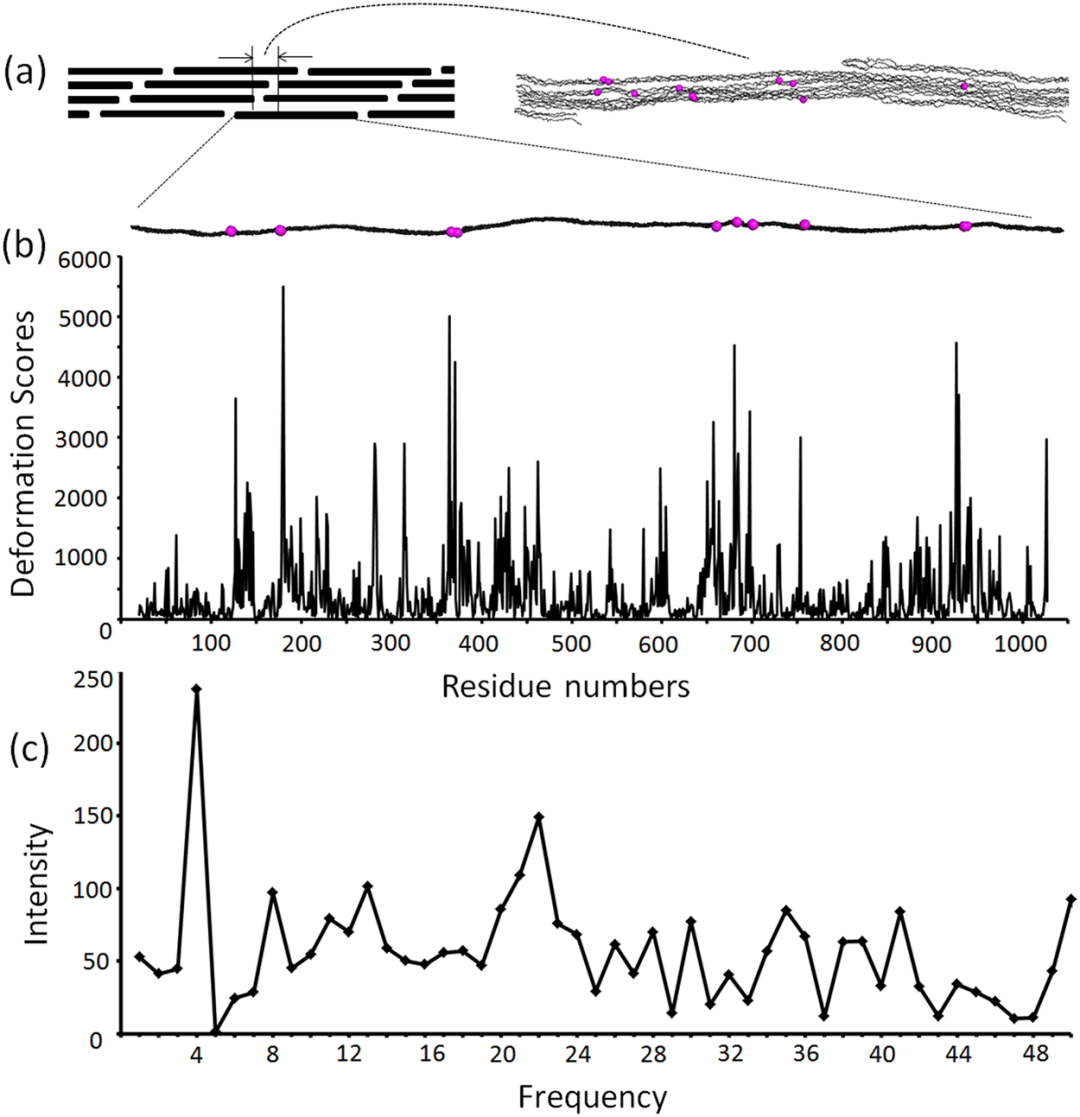
Highly deformed steps in natural collagen models^25^ and the fluctuation frequencies of the deformation scores found upon Fourier transformation. (**a**) Top left: a schematic representation of triple helix packing in the Type I collagen microfibril. Top right: an enlarged view of the *in situ* packing model. Bottom: an individual triple helix in the fibril. The 11 highly deformed steps (deformation scores ≥ 3000) highlighted with magenta spheres in the packing model and the individual triple helix. (**b**) The deformation scores are plotted against collagen sequence number. (**c**) Fluctuation frequency peaks of deformation scores are derived from Fourier transformation. The most significant frequency is 4, and the corresponding periodicity is 252 residues (periodicity = 1008 residues/frequency).

The steps with extremely high deformation scores (Δ*E* ≥ 3000) are distant from each other in terms of the collagen sequence. However, these steps cluster into three locations when mapped in an *in situ* packing unit of triple helical segments in natural collagen fibrils (Fig. 8(a)). The clustering of highly deformed steps suggests the interplay between the backbone deformation of single triple helices and the interhelical side chain interactions, both of which are important in collagen fibrillar assembly.

Fourier transformation analysis of the deformation scores along the long fibers reveals a prominent signal at a frequency of four (Fig. 8). To exclude artifacts from the low resolution, the atomic coordinates in the models were perturbed by the resolution value, 5 Å, and the major frequency was still four (Fig. S6). The fluctuation periodicity is consistent with the D-periodic banding (~67 nm) of collagen microfibrils seen with electron microscopy, slightly under one quarter of the full length of an uninterrupted triple helix^43^. This suggests that the morphology of the collagen fibrillar assembly might be rooted from the conformational fluctuation of collagen triple helices.

## Conclusions

Step parameters provide a way to assess the conformations of long thin helices such as collagen and DNA. These models provide a quantitative method to describe how collagen and DNA respond to ligand recognition. The conformational distortions may arise either directly from tight ligand-induced binding or result from multiple-domain cooperation.

At a larger scale, collagen and DNA deformability are expected to contribute to their natural higher-order packing morphologies. As a genetic information carrier, DNA assumes supercoiled conformations in nucleosomes in eukaryotic cells^44^. The coupling of Slide and Roll facilitates the wrapping of nucleosomal DNA around histone proteins^7^. Similarly, the ability of collagen to deform at specific sites allows it to form a near-crystalline lattice *in vivo*, as part of a hierarchical fiber. The step parameters and deformation scores can also be useful tools to extract oscillation modes from atomic-level molecular dynamic trajectories of collagen fibrillar assemblies. The elastic model may prove useful in parameterizing other multi-stranded helical biopolymers such as α-helical coiled coils or cross-β arrangements of amyloids.

## Electronic supplementary material

Parallels between DNA and collagen – comparing elastic models of the double and triple helix
