# Supplementary material for: Parallels between DNA and collagen – comparing elastic models of the double and triple helix
Source: Sci Rep. 2017 Oct 16;7:12802. doi: 10.1038/s41598-017-12878-3 (PMC5643560; doi:10.1038/s41598-017-12878-3)
Supplement: Supplementary file 1 — Parallels between DNA and collagen – comparing elastic models of the double and triple helix [file 41598_2017_12878_MOESM1_ESM.pdf]

Parallels between DNA and collagen – comparing elastic models of the double and triple helix

Fei Xu[a,1], Hong-Ning Zheng[a], Nicolas Clauvelin[b], Xiang-Jun Lu[c], Wilma K. Olson[b,1], Vikas Nanda[d,1]

[a]School of Biotechnology, Jiangnan University, 1800 Lihu Ave. Wuxi, Jiangsu 214122, China

[b]Department of Chemistry and Chemical Biology, Rutgers University, 610 Taylor Road, Piscataway, NJ 08854, USA

[c]Department of Biological Sciences, Columbia University, New York, NY10027, USA

[d]Center for Advanced Biotechnology and Medicine, Rutgers University, 679 Hoes Lane West, Piscataway, NJ 08854, USA

Supplemental Information

Methods

Calculation scheme of triple helical step parameters

The six step parameters relating collagen triplets are computed with respect to a middle reference frame using the matrix-based algorithm developed by El Hassan and Calladine (El Hassan and Calladine 1995), defined by Dickerson (Dickerson 1989) and implemented within the 3DNA suite of programs (Lu and Olson 2003) . The middle reference frame is set up by transforming the two Cα triangle reference frames into the middle one with a series of Euler-angle-based movements. For two Cα triangle reference frames, T_i and T_{i+1}, Γ is the dihedral angle between their *x,y*-planes. The T_i and T_{i+1}*x, y*-planes are rotated by $\Gamma/2$ and $-\Gamma/2$ around the plane intersection line to align their *z*-axes, and thereby locate the *z*-axis of the middle frame, T_{mst}. The rotation angle needed to align the *x*-axes (or *y*-axes) of the rotated T_i and T_{i+1}frame is defined as the absolute value of Twist, denoted as Ω, and the middle vector of these corresponding axes is set as the T_{mst}*x*-axis (or *y*-axis). The angle between the intersection line and the T_{mst}*y*-axis is denoted as ϕ. The projections of Γon theT_{mst}*x*- and *y*-axes with phase angle ϕ are defined as Tilt and Roll, respectively. The T_{mst} origin is the middle point between the T_i and T_{i+1} origins. The translational parameters, Shift, Slide, and Rise are the components of the vector between the T_i and T_{i+1} origins projected on the T_{mst}*x*-, *y*-, and *z*-axes, respectively.

The calculation scheme of the algorithm is summarized as follows:

$$T_{i+1} = \left[R_z(\frac{\Omega}{2} - \phi)R_y(\Gamma)R_z(\frac{\Omega}{2} + \phi)\right]T_i, \qquad \text{(S1)}$$

$$T_{mst} = \left[R_z(\frac{\Omega}{2} - \phi)R_y(\frac{\Gamma}{2})R_z(\phi)\right]T_i, \qquad \text{(S2)}$$

$$O_{i+1} = O_i + D_x x_{mst} + D_y y_{mst} + D_z z_{mst}. \qquad \text{(S3)}$$

Constructing schematic models of the triple helix and computing helical periodicity

Given a set of step parameters and a starting reference frame, a Cα-triangle reference frame is deduced with the reversed procedure mentioned above (Eq. (S1-S3)). The schematic triangular slab models in Fig. 2 are generated with modified code of the 3DNA software package (Lu and Olson 2008).

An averaged Cα-triangle shape of GP_2 and GP_0 is placed on the rebuilt reference frames in order to obtain Cα atom coordinates. As this is an ideally repeated model, the Gly Cα atom coordinates from four successive Cα-triangles are used to deduce triple-helical periodicity (Miyazawa 1961, Sugeta and Miyazawa 1967). The corresponding Gly Cα atoms are connected by three uniform pseudo-bonds. The cylindrical angle θ and displacement d of the triple helices are deduced from the pseudo bond length r, pseudo valence angle φ, and pseudo dihedral angleτ with Eqs. (S4) and (S5).

$$\sin\left(\frac{\theta}{2}\right) = \cos(\frac{\tau}{2})\sin(\frac{\varphi}{2}), \qquad \text{(S4)}$$

$$d\sin\left(\frac{\theta}{2}\right) = r\sin(\frac{\tau}{2})\sin(\theta/2). \quad \text{(S5)}$$

Data selection procedure

Forty well-resolved X-ray crystal structures of collagen were selected from the Protein Data Bank and listed at Table S1.Triple-helical steps located at N- and C-termini, which may not be well folded or whose conformation may be distorted by crystal lattice packing, are not included in the dataset. The total number of accepted steps is 864. Severely distorted steps with extreme values are eliminated by a “culling” procedure (Olson, Gorin et al. 1998). We observed distorted steps due to receptor binding and lattice packing. The values outside three root-mean-square deviations, 3σ, are considered to be outliers. The outliers are iteratively removed from the dataset until all the values are tightly clustered within the 3σ limit as a quasi-Gaussian distribution. The root-mean-square deviation, σ, of a dataset containing n samples is calculated as below:

$$\sigma = \sqrt{\frac{\sum_{i=1}^{n}(x_i - \bar{x})}{n}}, \quad \text{(S6)}$$

where $\bar{x}$ is the mean value over all of the n samples

Plotting an equi-potential contour of the elastic function

The scoring function of the six geometric parameters can be considered as an energy surface in six-dimensional space. When the multi-dimensional space is projected onto a two-dimensional plane of any two parameters, it forms an equi-potential contour, on which any point has the same deformation energy (Olson, Gorin et al. 1998). For any two of the six step parameters i, j, the equi-potential contour is expressed as below:

$$E = \frac{1}{2}f_{i,i}(\theta_i - \theta_i^0)^2 + \frac{1}{2}\left(f_{i,j} + f_{j,i}\right)(\theta_i - \theta_i^0)\left(\theta_j - \theta_j^0\right) + \frac{1}{2}f_{j,j}(\theta_j - \theta_j^0)^2, \qquad \text{(S7)}$$

This equation shows that the contour is an ellipse and its geometric properties are calculated as below:

Length of the two axes

$$L_1 = \sqrt{\left|\frac{2}{f_{i,i}}\right|}, \quad L_2 = \sqrt{\left|\frac{2}{f_{j,j}}\right|}, \qquad \text{(S8)}$$

Inclination angle of the ellipse

$$\varphi = \frac{1}{2}\tan^{-1}\left(\frac{f_{i,j}+f_{j,i}}{f_{i,i}-f_{j,j}}\right). \qquad \text{(S9)}$$

Here $f_{i,i}$ and $f_{j,j}$, diagonal terms of the force constant matrix F, are the force constants impeding deformations of individual parameters. The force constants, $f_{i,j}$ and $f_{j,i}$, measure the coupling between the ith and j^{th} parameters and are cross terms of the symmetric matrix, i.e., $f_{i,j} = f_{j,i}$.

If the inclination of an ellipse is 45±25° and the major/minor axis ratio is greater than 1.2, the corresponding parameters are defined as highly coupled.

Linear correlation coefficients

The linear correlation coefficients between step parameters are calculated as below.

$$r = \frac{\sum_{i=1}^{n}(X_i-\bar{X})(Y_i-\bar{Y})}{\sqrt{\sum_{i=1}^{n}(X_i-\bar{X})^2\sum_{i=1}^{n}(Y_i-\bar{Y})^2}}, \qquad \text{(S10)}$$

where X_i and Y_i represent corresponding data in two groups, *n* is the number of samples, $\bar{X}$ and $\bar{Y}$ are the mean values.

Fourier Transformation analysis of natural collagen type I

All-atom models of collagen, kindly provided by Dr. Orgel and Dr. Varma, were constructed from fiber diffraction data (PDB ID: 3HR2) with THeBuScr, a collagen rebuilding tool and optimized by CNS 1.1 (Brünger, Adams et al. 1998, Rainey and Goh 2004, Orgel, Irving et al. 2006, Varma, Botlani et al. 2015). The step parameters and deformation scores of the 1008 steps contained in the collagenous region (chains A and C 17-1025, chain B 9-1017) were computed and analyzed with the fast Fourier Transformation function of the Matlab software package. The highest frequency peak is associated with the most predominant periodicity.

Calculation of DNA deformation scores

The step parameters of DNA were calculated with the 3DNA software package (Lu and Olson 2008). The DNA deformation scores were computed with knowledge-based

elastic functions (Olson, Gorin et al. 1998) and the force constants were derived from an updated dataset of protein-DNA interactions (Balasubramanian, Xu et al. 2009).

References

Balasubramanian, S., F. Xu and W. K. Olson (2009). "DNA sequence-directed organization of chromatin: structure-based computational analysis of nucleosome-binding sequences." Biophysical Journal 96(6): 2245-2260.

Brünger, A. T., P. D. Adams, G. M. Clore, W. L. Delano, P. Gros, R. W. Grosse-Kunstleve, J. S. Jiang, J. Kuszewski, M. Nilges and N. S. Pannu (1998). "Crystallography & NMR System : A New Software Suite for Macromolecular Structure Determination." Acta Crystallographica 54(Pt 5): 905-921.

Dickerson, R. E. (1989). "Definitions and nomenclature of nucleic acid structure components." Nucleic Acids Research 17(5): 1797-1803.

El Hassan, M. A. and C. R. Calladine (1995). "The assessment of the geometry of dinucleotide steps in double-helical DNA; a new local calculation scheme." Journal of Molecular Biology 251(5): 648-664.

Lu, X.-J. and W. K. Olson (2008). "3DNA: a versatile, integrated software system for the analysis, rebuilding and visualization of three-dimensional nucleic-acid structures." Nat. Protocols 3(7): 1213-1227.

Lu, X. J. and W. K. Olson (2003). "3DNA: a software package for the analysis, rebuilding and visualization of three-dimensional nucleic acid structures." Nucleic Acids Research 31(17): 5108-5121.

Miyazawa, T. (1961). "Molecular vibrations and structure of high polymers. II. Helical parameters of infinite polymer chains as functions of bond lengths, bond angles, and internal rotation angles." Journal of Polymer Science 55(161): 215-231.

Olson, W. K., A. A. Gorin, X.-J. Lu, L. M. Hock and V. B. Zhurkin (1998). "DNA sequence-dependent deformability deduced from protein–DNA crystal complexes." Proceedings of the National Academy of Sciences of the United States of America 95(19): 11163-11168.

Orgel, J. P., T. C. Irving, A. Miller and T. J. Wess (2006). "Microfibrillar structure of type I collagen in situ." Proceedings of the National Academy of Sciences of the United States of America 103(24): 9001-9005.

Rainey, J. K. and M. C. Goh (2004). "An interactive triple-helical collagen builder." Bioinformatics 20(15): 2458-2459.

Sugeta, H. and T. Miyazawa (1967). "General method for calculating helical parameters of polymer chains from bond lengths, bond angles, and internal-rotation angles." Biopolymers 5(7): 673-679.

Varma, S., M. Botlani, J. R. Hammond, H. L. Scott, J. P. R. O. Orgel and J. D. Schieber (2015). "Effect of intrinsic and extrinsic factors on the simulated D-band length of type I collagen." Proteins Structure Function & Bioinformatics 83(10): 1800-1812.

Young, H. D. (1962). Statistical Treatment of Experimental Data. New York, McGraw-Hill.

Supplementary figures and Tales:

Table S1. Collagen structures used to generate knowledge-based potentials.

No.	PDB ID	Description*	Resolution (Å)	Literature Citation
1	1WZB	$(OOG)_{10}$ collagen peptide.	1.50	(Kawahara, Nishi et al. 2005)
2	3DMW	Cystine knotted collagen containing human type III collagen fragment Gly991-Gly1032. The sequence is IGPOGPRGNRGERGSEGSOGHOGMOGPOGAOGPC.	2.30	(Boudko, Engel et al. 2008)
3	2CUO	$(PPG)_9$ collagen peptide.	1.33	(Hongo, Noguchi et al. 2005)
4	1YM8	$(GOO)_9$ collagen peptide.	1.55	(Schumacher, Mizuno et al. 2005)
5	1CGD	$(POG)_4AOG(POG)_5$ collagen peptide.	1.85	(Bella, Brodsky et al. 1995)
6	2D3H	$(PPG)_4OOG(PPG)_4$ collagen peptide.	1.22	(Okuyama, Hongo et al. 2009)
7	2D3F	$(PPG)_4POG(PPG)_4$ collagen peptide.	1.26	(Okuyama, Hongo et al. 2009)
8	1BKV	$(GPO)_3GITGARGLA(GPO)_3$ collagen peptide.	2.00	(Kramer, Bella et al. 1999)
9	1ITT	GPPGPPG collagen peptide.	1.00	(Hongo, Nagarajan et al. 2001)
10	2DRT	$(POG)_4LOG(POG)_5$ collagen peptide.	1.60	(Okuyama, Narita et al. 2007)
11	2DRX	$(POG)_4LOG(POG)_4$ collagen peptide.	1.40	(Okuyama, Narita et al. 2007)
12	1K6F	$(PPG)_{10}$ collagen peptide.	1.30	(Berisio, Vitagliano et al. 2002)
13	2G66	$(GPO)_3(GOO)_2(GPO)_4$ collagen peptide.	1.80	(Schumacher, Mizuno et al. 2006)
14	1NAY	$(GPP)_{10}$ peptide linked to trimeric domain foldon from	2.60	(Stetefeld, Frank et

		bacteriophage T4 fibritin with a short Gly-Ser linker.		al. 2003)
15	1V7H	GPOGPOG collagen peptide.	1.25	(Okuyama, Hongo et al. 2004)
16	2WUH	DDR2 (Discoidin Domain Receptor) and collagen binding complex with the collagen sequence (GPO)$_3$PRGQOGVNleGFO(GPO)$_2$G, where Nle is norleucine.	1.60	(Carafoli, Bihan et al. 2009)
17	1QSU	(POG)$_4$EKG(POG)$_5$ collagen peptide.	1.75	(Kramer, Venugopal et al. 2000)
18	2V53	Collagen-SPARC binding complex containing (GPO)$_2$GPSGPRGQOGVMGFOGAO collagen peptides.	3.20	(Hohenester, Sasaki et al. 2008)
19	1X1K	(PPG)$_4$PXG(PPG)$_4$ collagen peptide, where X is non-natural isomer 4S-hydroxy-proline.	1.10	(Jiravanichanun, Hongo et al. 2005)
20	3P46	(GPO)$_2$GLOGEA(GPO)$_2$ collagen peptide.	1.70	(Byrne, McEwan et al. 2011)
21	1EI8	Collagen peptide (POG)$_4$PG(POG)$_5$ with a sequence disruption.	2.00	(Bella, Liu et al. 2006)
22	3T4F	Collagen peptide (POG)$_3$PKGEOG(POG)$_3$.	1.68	(Fallas, Dong et al. 2012)
23	3U29	Collagen peptide (POG)$_3$PKGDOG(POG)$_3$.	2.00	(Fallas, Dong et al. 2012)
24	3POB	Mannan-binding lectin and collagen binding complex with the collagen sequence (GPO)$_3$GKL(GPO)$_4$.	1.80	(Gingras, Girija et al. 2011)
25	2F6A	*Staphylococcus aureus* CAN-collagen binding complex with the collagen sequence (GPO)$_4$GPRGRT(GPO)$_4$GP.	3.29	(Zong, Xu et al. 2005)
26	5CJB	Human osteoclast associated receptor-collagen binding complex with the collagen sequence (GPO)$_3$GPOGPAGFO(GPO)$_2$G	2.40	(Haywood, Qi et al. 2016)
27	5EIV	Human osteoclast associated receptor-collagen binding complex with the collagen sequence (GPP)$_3$GPAGFP(GPP)$_2$	2.41	(Zhou, Hinerman et al. 2015)
28	3WN8	(POG)$_3$PRG(POG)$_4$ collagen peptide	1.45	(Okuyama, Haga et al. 2014)
29	4BKL	Arthritogenic antibody M2139-collagen binding complex with the collagen sequence (GPP)$_5$GMPGERGAAGIAGPK(GPP)$_2$G	3.25	(Bruno, Doreen et al. 2014)
30	4LOR	CUB1-EGF-CUB2 complexed with (GPP)4GKL(GPP)4 collagen	2.50	(Venkatraman Girija,

		peptide		Gingras et al. 2013)
31	3ZHA	Hsp47 SERPINH1-conllagen complex with the collagen sequence $(PPG)_2PTGPR(GPP)_2$	2.55	(Christine, Gebauer et al. 2012)
32	4AXY	$(PPG)_2PTGPR(GPP)_2$ collagen peptide	1.24	(Christine, Gebauer et al. 2012)
33	4GYX	$(GPP)_2GPRGQPGVMGFP(GPP)_2GPCCGGV$ collagen peptide	1.49	(Boudko and Hans Peter 2012)
34	4AU2	Hsp47 SERPINH1-conllagen complex with the collagen sequence PPGPPGPRGPPGPP	2.30	(Christine, Gebauer et al. 2012)
35	2Y5T	Pathogenic autoantibody CIIC1-collagen complex with the collagen sequence $(GPP)_5GARGLTGRPGDA(GPP)_2G$	2.20	(Dobritzsch, Lindh et al. 2011)
36	3ADM	Collagen peptide $(PPG)_4OSG(PPG)4$	1.18	(Okuyama, Miyama et al. 2011)
37	3P46	Collagen peptide $(GPP)_2GLPGEA(GPP)_2$	1.70	(Byrne, McEwan et al. 2011)
38	3ABN	Collagen peptide $(PPG)_4ODG(PPG)_4$	1.02	(Okuyama, Miyama et al. 2011)
39	3A1H	Collagen peptide $(PPG)_4OTG(PPG)_4$	1.08	(Okuyama, Miyama et al. 2011)
40	3POD	Collagen peptide $(GPP)_3GKL(GPP)_4$	1.50	(Gingras, Girija et al. 2011)

* O is hydroxy-proline.

Bella, J., B. Brodsky and H. M. Berman (1995). "Hydration structure of a collagen peptide." Structure **3**(9): 893-906.

Bella, J., J. Liu, R. Kramer, B. Brodsky and H. M. Berman (2006). "Conformational Effects of Gly–X–Gly Interruptions in the Collagen Triple Helix." Journal of Molecular Biology **362**(2): 298-311.

Berisio, R., L. Vitagliano, L. Mazzarella and A. Zagari (2002). "Crystal structure of the collagen triple helix model [(Pro-Pro-Gly)10]3." Protein Science **11**(2): 262-270.

Boudko, S. P., J. Engel, K. Okuyama, K. Mizuno, H. P. Bächinger and M. A. Schumacher (2008). "Crystal Structure of Human Type III Collagen Gly991–Gly1032 Cystine Knot-containing Peptide Shows Both 7/2 and 10/3 Triple Helical Symmetries." Journal of Biological Chemistry **283**(47): 32580-32589.

Boudko, S. P. and B. C. Hans Peter (2012). "The NC2 domain of type IX collagen determines the chain register of the triple helix." Journal of Biological Chemistry **287**(53): 44536-44545.

Bruno, R., D. Doreen, G. Changrong, E. Diana, X. Bingze, L. Ingrid, F. R. Michael, U. Hüseyin, N. Kutty Selva and S. Gunter (2014). "Epitope-specific antibody response is controlled by immunoglobulin V(H) polymorphisms." Journal of Experimental Medicine **211**(3): 405-411.

Byrne, C., P. A. McEwan, J. Emsley, P. M. Fischer and W. C. Chan (2011). "End-stapled homo and hetero collagen triple helices: a click chemistry approach." Chemical Communications **47**(9).

Byrne, C., P. A. McEwan, J. Emsley, P. M. Fischer and W. C. Chan (2011). "End-stapled homo and hetero collagen triple helices: a click chemistry approach." Chemical Communications **47**(9): 2589-2591.

Carafoli, F., D. Bihan, S. Stathopoulos, A. D. Konitsiotis, M. Kvansakul, R. W. Farndale, B. Leitinger and E. Hohenester (2009). "Crystallographic Insight into Collagen Recognition by Discoidin Domain Receptor 2." Structure **17**(12): 1573-1581.

Christine, W., J. M. Gebauer, B. Elena, R. Sabrina, Z. Frank, D. G. Cord, L. Tosso and B. Ulrich (2012). "Molecular basis for the action of the collagen-specific chaperone Hsp47/SERPINH1 and its structure-specific client recognition." Proceedings of the National Academy of Sciences **109**(33): 13243-13247.

Dobritzsch, D., I. Lindh, H. Uysal, K. S. Nandakumar, H. Burkhardt, G. Schneider and R. Holmdahl (2011). "Crystal structure of an arthritogenic anticollagen immune complex." Arthritis & Rheumatism **63**(12): 3740-3748.

Fallas, J. A., J. Dong, Y. J. Tao and J. D. Hartgerink (2012). "Structural Insights into Charge Pair Interactions in Triple Helical Collagen-like Proteins." Journal of Biological Chemistry **287**(11): 8039-8047.

Gingras, A., U. V. Girija, A. Keeble, R. Panchal, D. Mitchell, P. E. Moody and R. Wallis (2011). "Structural Basis of Mannan-Binding Lectin Recognition by Its Associated Serine Protease MASP-1: Implications for Complement Activation." Structure **19**(11): 1635-1643.

Gingras, Alexandre R., Umakhanth V. Girija, Anthony H. Keeble, R. Panchal, Daniel A. Mitchell, Peter C. E. Moody and R. Wallis (2011). "Structural Basis of Mannan-Binding Lectin Recognition by Its Associated Serine Protease MASP-1: Implications for Complement Activation." Structure **19**(11): 1635-1643.

Haywood, J., J. Qi, C. C. Chen, G. Lu, Y. Liu, J. Yan, Y. Shi and G. F. Gao (2016). "Structural basis of collagen recognition by human osteoclast-associated receptor and design of osteoclastogenesis inhibitors." Proceedings of the National Academy of Sciences.

Hohenester, E., T. Sasaki, C. Giudici, R. W. Farndale and H. P. Bächinger (2008). "Structural basis of sequence-specific collagen recognition by SPARC." Proceedings of the National Academy of Sciences**105**(47): 18273-18277.

Hongo, C., V. Nagarajan, K. Noguchi, S. Kamitori, K. Okuyama, Y. Tanaka and N. Nishino (2001). "Average crystal structure of (Pro-Pro-Gly)9 at 1.0 angstroms resolution." Plym. J.**33**: 812.

Hongo, C., K. Noguchi, K. Okuyama, Y. Tanaka and N. Nishino (2005). "Repetitive Interactions Observed in the Crystal Structure of a Collagen-Model Peptide, [(Pro-Pro-Gly)9]3." Journal of Biochemistry**138**(2): 135-144.

Jiravanichanun, N., C. Hongo, G. Wu, K. Noguchi, K. Okuyama, N. Nishino and T. Silva (2005). "Unexpected Puckering of Hydroxyproline in the Guest Triplets, Hyp-Pro-Gly and Pro-alloHyp-Gly Sandwiched between Pro-Pro-Gly Sequence." ChemBioChem**6**(7): 1184-1187.

Kawahara, K., Y. Nishi, S. Nakamura, S. Uchiyama, Y. Nishiuchi, T. Nakazawa, T. Ohkubo and Y. Kobayashi (2005). "Effect of Hydration on the Stability of the Collagen-like Triple-Helical Structure of [4(R)-Hydroxyprolyl-4(R)-hydroxyprolylglycine]10‡." Biochemistry**44**(48): 15812-15822.

Kramer, R. Z., J. Bella, P. Mayville, B. Brodsky and H. M. Berman (1999). "Sequence dependent conformational variations of collagen triple-helical structure." Nat Struct Mol Biol**6**(5): 454-457.

Kramer, R. Z., M. G. Venugopal, J. Bella, P. Mayville, B. Brodsky and H. M. Berman (2000). "Staggered molecular packing in crystals of a collagen-like peptide with a single charged pair." Journal of Molecular Biology**301**(5): 1191-1205.

Okuyama, K., M. Haga, K. Noguchi and T. Tanaka (2014). "Preferred side-chain conformation of arginine residues in a triple-helical structure." Biopolymers**101**(10): 1000–1009.

Okuyama, K., C. Hongo, R. Fukushima, G. Wu, H. Narita, K. Noguchi, Y. Tanaka and N. Nishino (2004). "Crystal structures of collagen model peptides with Pro-Hyp-Gly repeating sequence at 1.26 Å resolution: Implications for proline ring puckering." Peptide Science**76**(5): 367-377.

Okuyama, K., C. Hongo, G. Wu, K. Mizuno, K. Noguchi, S. Ebisuzaki, Y. Tanaka, N. Nishino and H. P. and Bächinger (2009). "High-resolution structures of collagen-like peptides [(Pro-Pro-Gly)4-Xaa-Yaa-Gly-(Pro-Pro-Gly)4]: Implications for triple-helix hydration and Hyp(X) puckering." Biopolymers**91**: 361–372.

Okuyama, K., K. Miyama, T. Morimoto, K. Masakiyo, K. Mizuno and H. P. Bächinger (2011). "Stabilization of triple-helical structures of collagen peptides containing a Hyp-Thr-Gly, Hyp-Val-Gly, or Hyp-Ser-Gly sequence." Biopolymers**95**(9): 628-640.

Okuyama, K., H. Narita, T. Kawaguchi, K. Noguchi, Y. Tanaka and N. Nishino (2007). "Unique side chain conformation of a leu residue in a triple-helical structure." Biopolymers**86**(3): 212-221.

Schumacher, M., K. Mizuno and H. P. Bächinger (2005). "The Crystal Structure of the Collagen-like Polypeptide (Glycyl-4(R)-hydroxyprolyl-4(R)-hydroxyprolyl)9 at 1.55 Å Resolution Shows Up-puckering of the Proline Ring in the Xaa Position." Journal of Biological Chemistry**280**(21): 20397-20403.

Schumacher, M. A., K. Mizuno and H. P. Bächinger (2006). "The Crystal Structure of a Collagen-like Polypeptide with 3(S)-Hydroxyproline Residues in the Xaa Position Forms a Standard 7/2 Collagen Triple Helix." Journal of Biological Chemistry**281**(37): 27566-27574.

Stetefeld, J., S. Frank, M. Jenny, T. Schulthess, R. A. Kammerer, S. Boudko, R. Landwehr, K. Okuyama and J. Engel (2003). "Collagen Stabilization at Atomic Level: Crystal Structure of Designed (GlyProPro)10foldon." Structure**11**(3): 339-346.

Venkatraman Girija, U., A. R. Gingras, J. E. Marshall, R. Panchal, M. A. Sheikh, P. Gál, W. J. Schwaeble, D. A. Mitchell, P. C. E. Moody and R. Wallis (2013). "Structural basis of the C1q/C1s interaction and its central role in assembly of the C1 complex of complement activation." Proceedings of the National Academy of Sciences of the United States of America**110**(34): 13916-13920.

Zhou, L., J. M. Hinerman, M. Blaszczyk, J. L. Miller, D. G. Conrady, A. D. Barrow, D. Y. Chirgadze, D. Bihan, R. W. Farndale and A. B. Herr (2015). "Structural basis for collagen recognition by the immune receptor OSCAR." Blood**127**.

Zong, Y., Y. Xu, X. Liang, D. R. Keene, A. Hook, S. Gurusiddappa, M. Hook and S. V. L. Narayana (2005). "A /`Collagen Hug/' Model for Staphylococcus aureus CNA binding to collagen." EMBO J**24**(24): 4224-4236.

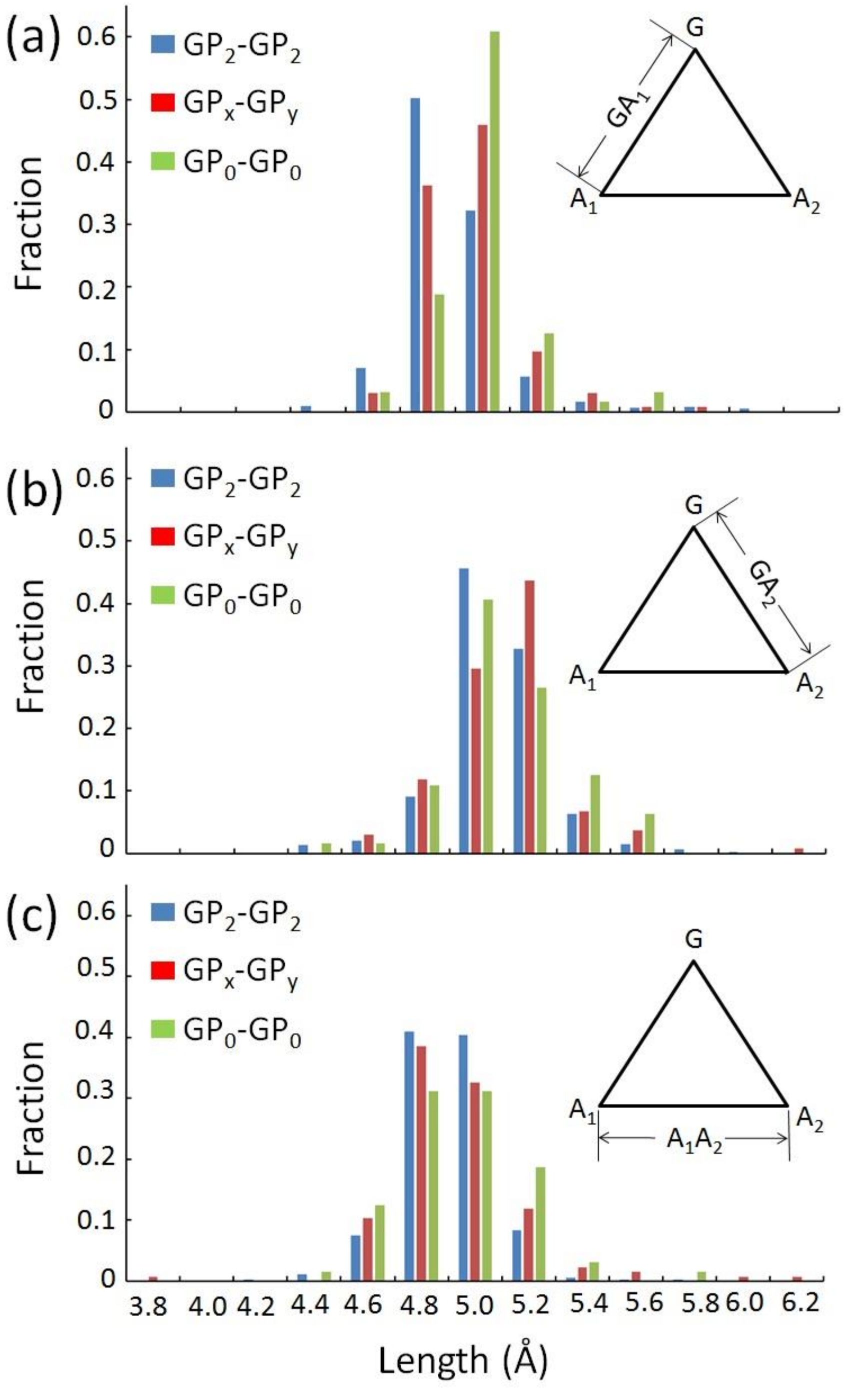

Figure S1. The side length of Cα triangles of the three imino-groups GP_2-GP_2, GP_x-GP_y ($x<2$ or $y<2$) and GP_0-GP_0, where subscripts in GP_2, GP_1 and GP_0 are the numbers of either Pro or Hyp in a Gly/X/Y triad.

Table S2. Helical parameters of the average structure models.

Models	Superhelical Twist (°)	Steps per turn	Superhelical Rise (Å)	Superhelical pitch (Å)
GP_2-GP_2	-103.08	3.49	2.93	10.23
GP_x-GP_y (x<2 or y<2)	-104.82	3.43	2.96	10.16
GP_0-GP_0	-107.25	3.36	3.04	10.19

* Subscripts are the numbers of imino (Pro/Hyp) in aGly/X/Y triad

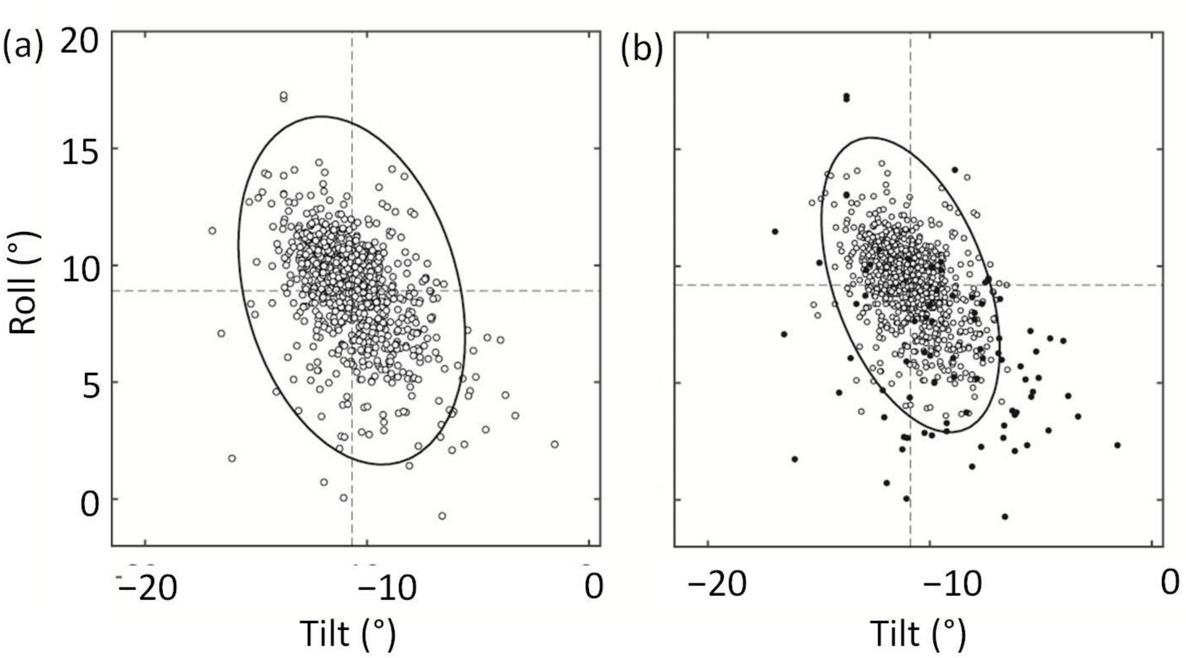

Figure S2. Scatter plots of Tilt-Roll and the derived energy contours (a) before, and (b) after culling procedure. The data points, which are excluded with the culling procedure, are shown as filled black dots.

Table S3. Force constants impeding triangle steps deformation of collagen triple helix are listed in (a). Because the energies are in $k_B T$, the units of the force constants for angular, translational and angular-translational deformation are $k_B T\text{deg}^{-2}$, $k_B T Å^{-2}$, $k_B T\text{deg}^{-1}Å^{-1}$. The shape and orientation of derived elliptical contours show the coupling between the parameters in Fig. 3. Inclination angles and axis ratios of the elliptical contours are listed in (b). If inclination of an ellipsis is 45±25° and the long/short axis ratio is greater than 1.2, the corresponding parameters are defined as highly coupled and highlighted in bold.

(a)

Relationship	GP_2-GP_2*	GP_x-GP_y (x<2 or y<2)	GP_0-GP_0
Shift-Shift	539.66	536.62	444.86
Shift-Slide	**–278.46**	**–219.25**	**–178.07**
Shift-Rise	**–746.38**	**–704.24**	**–650.89**
Shift-Tilt	–21.1	–16.61	–15.46
Shift-Roll	–8.44	–7.4	–6.34
Shift-Twist	–24.03	–20.21	–13.09
Slide-Slide	228.69	146.60	115.60
Slide-Rise	411.47	321.56	304.11
Slide-Tilt	11.49	6.43	4.49
Slide-Roll	1.7	1.07	1.62
Slide-Twist	14.26	8.39	4.12
Rise-Rise	1452.36	1199.75	1189.51
Rise-Tilt	27.3	20.87	16.46
Rise-Roll	12.88	10.36	9.69
Rise-Twist	27.6	22.44	13.13
Tilt-Tilt	1.75	1.14	1.15
Tilt-Roll	**0.5**	**0.3**	0.21
Tilt-Twist	**1.24**	**0.85**	**0.81**
Roll-Roll	0.69	0.47	0.42
Roll-Twist	0.28	0.15	0.11
Twist-Twist	1.47	1.11	0.72

* Subscripts are the numbers of imino (Pro/Hyp) in a Gly/X/Y triad

(b)

Relationship	GP_2-GP_2*		GP_x-GP_y (x<2 or y<2)		GP_0-GP_0	
	Inclination angle (°)	Axis ratio	Inclination angle (°)	Axis ratio	Inclination angle (°)	Axis ratio
Shift-Slide	**59.6**	**3.28**	**65.8**	**3.63**	**66.4**	**3.72**
Shift-Rise	**29.3**	**3.93**	**32.4**	**4.28**	**30.1**	**4.82**
Shift-Tilt	87.8	24.18	88.2	29.22	88.0	26.96
Shift-Roll	89.1	31.19	89.2	37.97	89.2	36.73
Shift-Twist	87.5	36.72	87.8	39.4	88.3	36.62
Slide-Rise	17.0	3.91	15.7	4.79	14.8	5.98
Slide-Tilt	87.1	13.99	87.5	13.07	87.8	10.9
Slide-Roll	89.6	18.41	89.6	17.72	89.2	17.06
Slide-Twist	86.4	19.89	86.7	15.32	88.0	14.25
Rise-Tilt	88.9	34.27	89.0	39.21	89.2	35.9
Rise-Roll	89.5	50.35	89.5	55.82	89.5	59.05
Rise-Twist	88.9	39.16	88.9	41.77	89.4	45.57
Tilt-Roll	**68.5**	**1.99**	**69.2**	**1.86**	75.2	1.82
Tilt-Twist	**48.2**	**2.79**	**45.6**	**2.7**	**52.5**	**4.31**
Roll-Twist	17.7	1.62	13.0	1.61	18.7	1.41

*Subscripts are the numbers of imino (Pro/Hyp) in a Gly/X/Y triad

Table S4. Linear correlation coefficients of the step parameters in three imino-content groups. The correlation values at the 0.001 significance level are approximately 0.35 for sample number greater than 100, and 0.5 for sample number greater than 40, respectively (Young 1962). There are 642, 180 and 42 data points in GP_2-GP_2, GP_x-GP_y(x<2 or y<2) and GP_0-GP_0, respectively. The coefficients representing highly correlated step parameters are highlighted in bold.

Relationship	GP_2-GP_2*	GP_x-GP_y (x<2 or y<2)	GP_0-GP_0
Shift-Slide	–0.1	0.19	–0.04
Shift-Rise	**0.67**	**0.69**	**0.83**
Shift-Tilt	–0.16	–0.33	–0.39
Shift-Roll	0.2	0.42	0.33
Shift-Twist	**0.74**	**0.68**	**0.72**
Slide-Rise	–**0.36**	–**0.35**	–0.49
Slide-Tilt	0.13	–0.11	0.19
Slide-Roll	**0.39**	0.24	0.26
Slide-Twist	–**0.41**	–0.17	–0.16
Rise-Tilt	–0.1	–0.24	–0.47
Rise-Roll	–0.11	0.11	0.05
Rise-Twist	**0.51**	**0.53**	**0.63**
Tilt-Roll	–0.33	–**0.39**	–0.32
Tilt-Twist	–**0.54**	–**0.6**	–**0.78**
Roll-Twist	0.15	**0.49**	0.34

*Subscripts are the numbers of imino (Pro/Hyp) in a Gly/X/Y triad

Table S5. Average values of step parameters from the 'culled' dataset. These values are used as 'equilibrium' states, θ_0, in Equation (1) to compute deformation energy scores.

Step	Step number	Shift (Å)	Slide (Å)	Rise (Å)	Tilt (°)	Roll (°)	Twist (°)
GP_2-GP_2*	642	−4.25±0.18	0.84±0.17	3.25±0.07	−11.14±1.51	9.46±1.94	−101.72±2.84
GP_x-GP_y (x<2 or y<2)	180	−4.40±0.20	0.75±0.21	3.14±0.15	−9.73±1.84	7.65±2.19	−104.25±3.24
GP_0-GP_0	42	−4.56±0.24	0.75±0.21	3.14±0.15	−7.89±2.63	5.97±2.25	−107.48±3.86

* Subscripts are the numbers of imino (Pro/Hyp) in a Gly/X/Y triad

Table S6. The mean values and standard deviation of the step parameters of natural collagen type I.

Step number	Shift (Å)	Slide (Å)	Rise (Å)	Tilt (°)	Roll (°)	Twist (°)
1008	−3.37±0.93	0.65±0.91	3.03±0.47	−8.62±8.72	7.13±10.07	−103.20±9.62

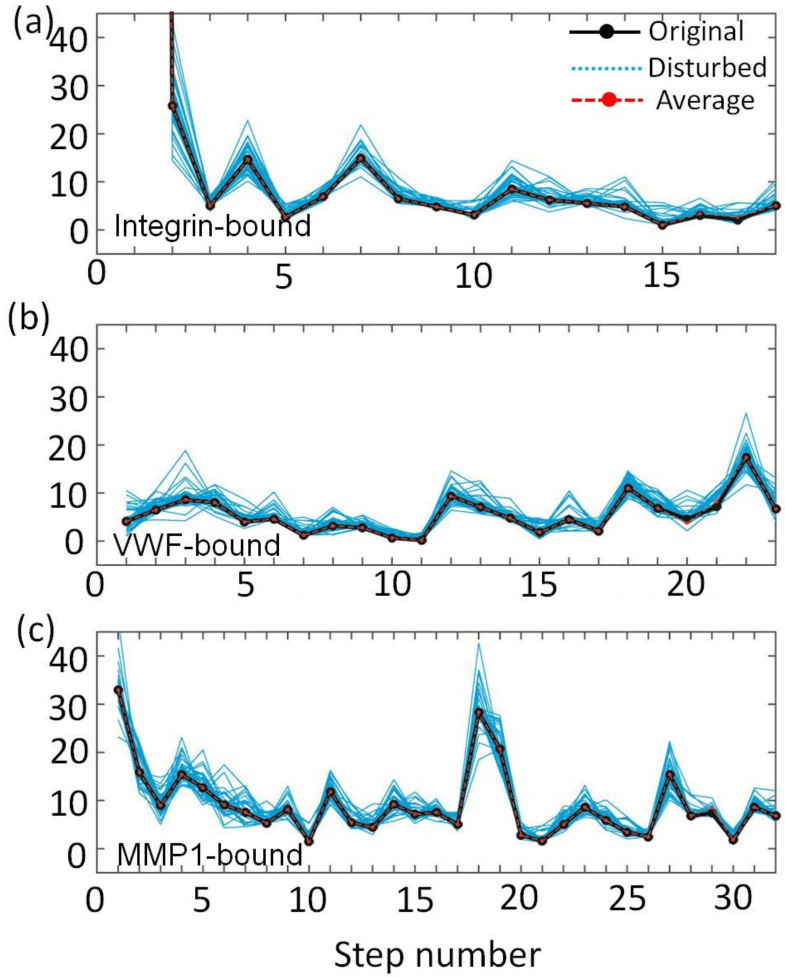

Figure S3. Deformation scores of (a) integrin-bound, (b) vWF-bound, and (c) MMP1-bound collagen structures (PDB ID: 1DZI, 4AUO, 4DMU) with the atom coordinates randomly disturbed with 0.1 Å compared to the original ones.

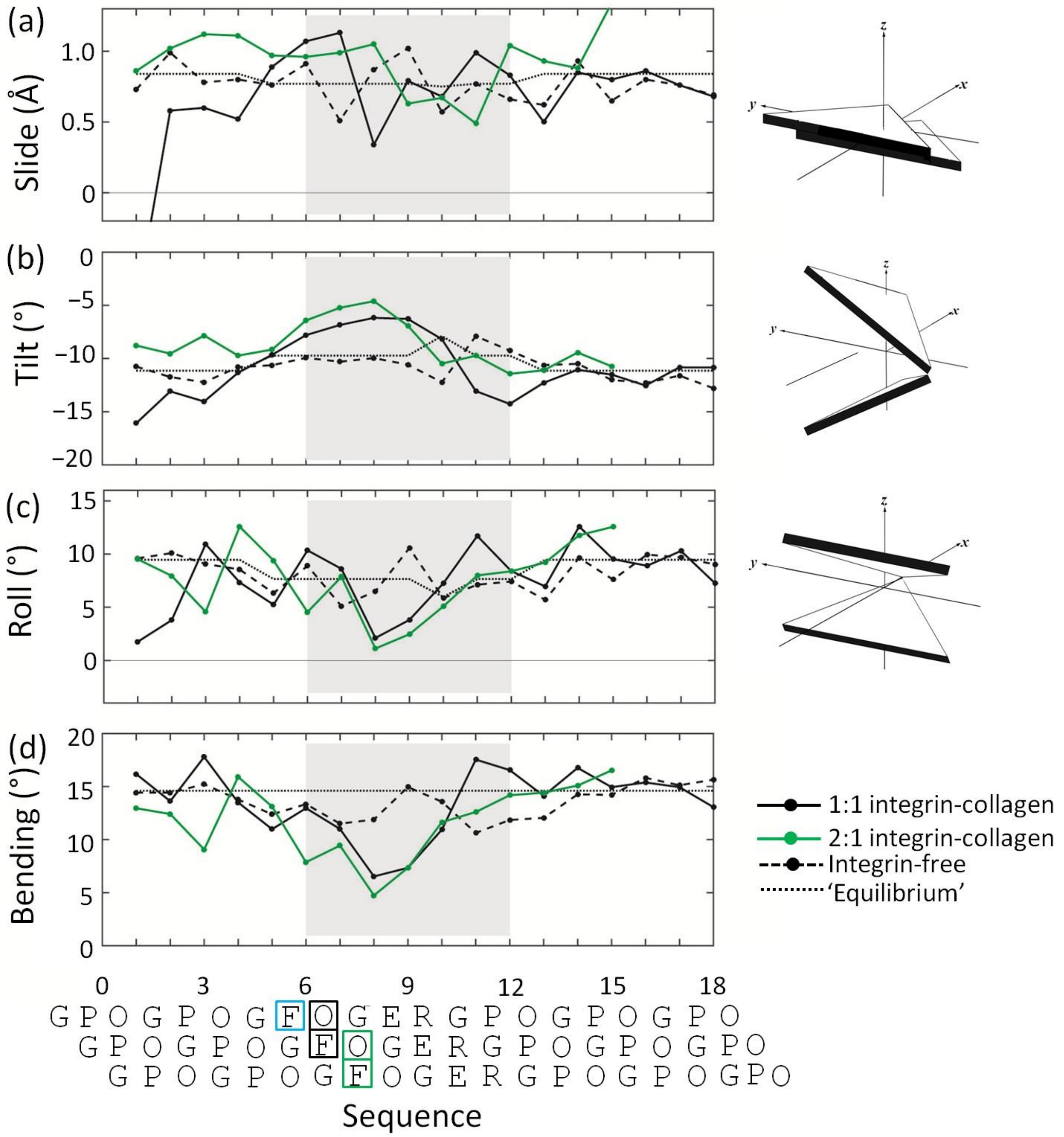

Figure S4. (a) Slide, (b) Tilt, (c) Roll, and (d) $\sqrt{\mathrm{Roll}^2 + \mathrm{Tilt}^2}$ (bending) of integrin-bound complexes (PDB: 1DZI, 4BJ3) and integrin-free (DPB: 1Q7D) triple helices with the same sequence (13, 33, 34). The mean step parameter values, or 'equilibrium states' are listed in Table S5.The integrin-bound region is shaded in grey. The sequences are aligned by step number. The Phe and Hyp residues contacted by integrin Asn154 and Leu296 are framed by color-coded rectangles: black for the 1:1 complex; green for integrin molecule A; light blue for integrin molecule B (contact only to Phe).

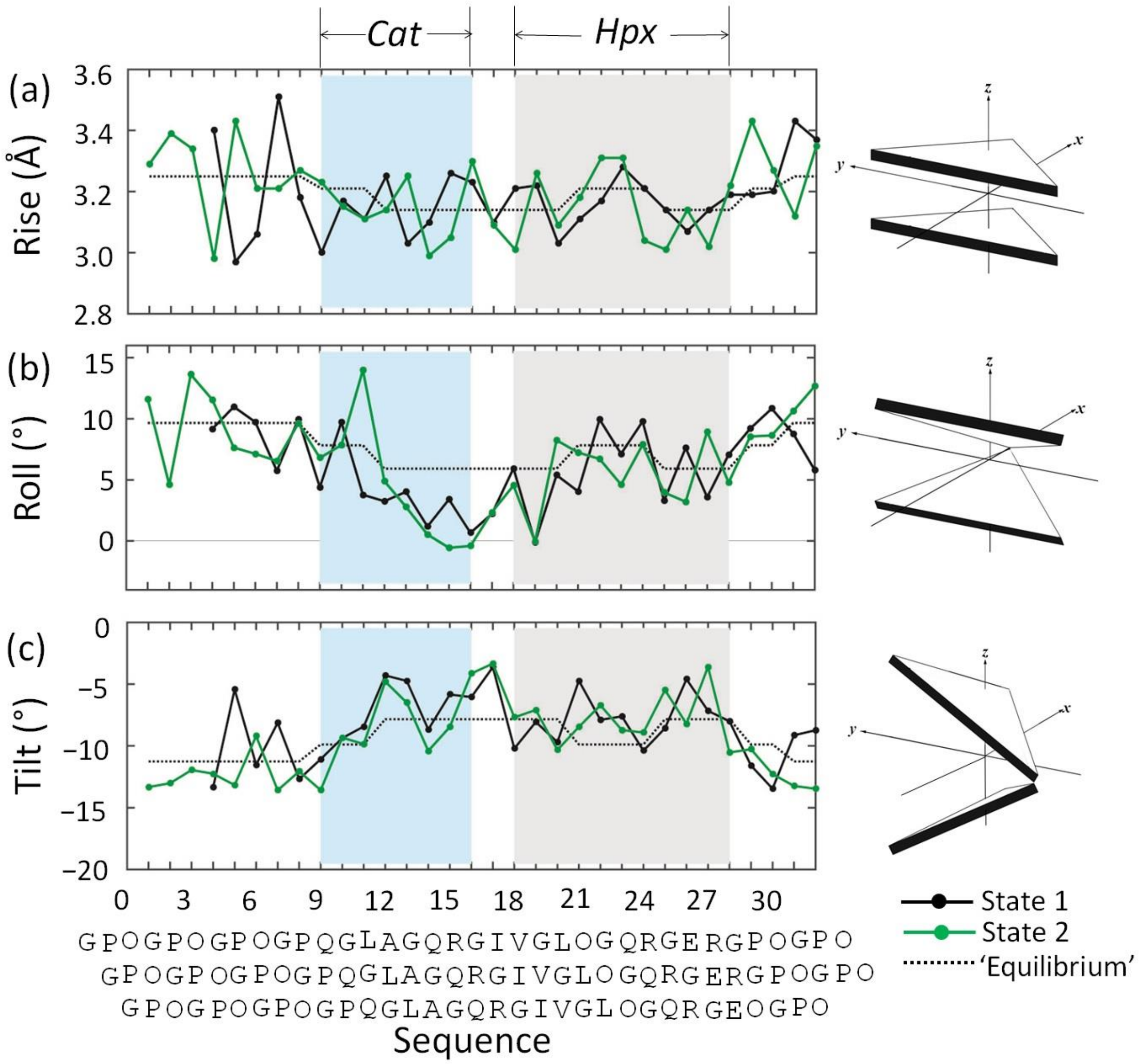

Figure S5. (a) Rise, (b) Roll, and (c) Tilt of the two states in integrin matrix metalloproteinase 1(MMP1)-bound triple helix structures (PDB: 4AUO). The mean step parameter values, or 'equilibrium states' are listed in Table S3. The sequences bound by MMP1 N-terminal catalytic (*Cat*) and C-terminal hemopexin (*Hpx*) domains were shaded in light cyan and grey, respectively. The sequences are aligned along the step number.

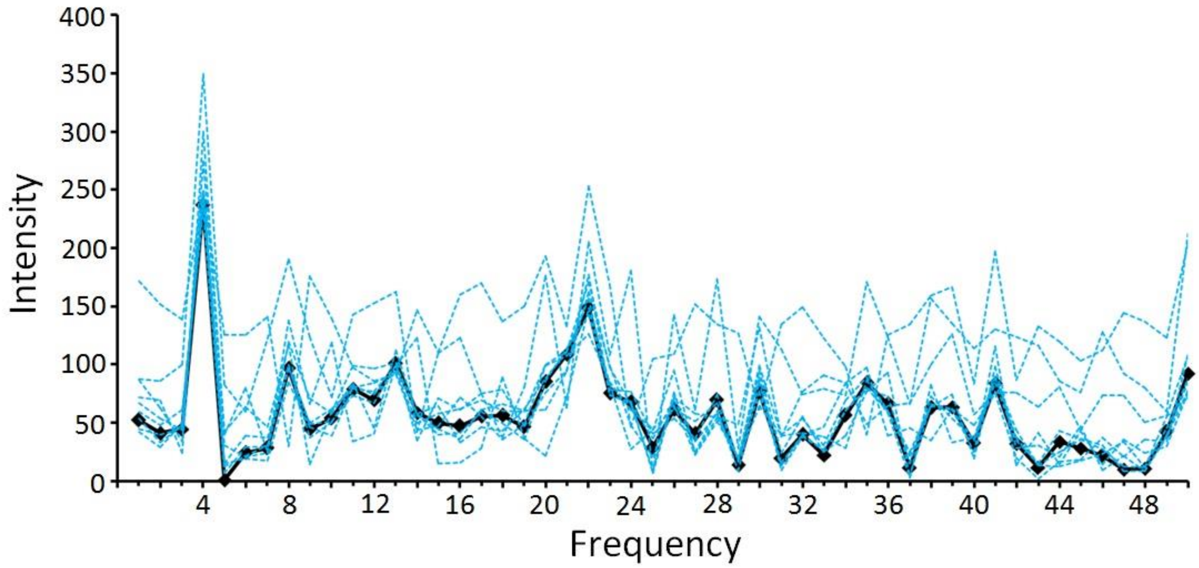

Figure S6. Sensitivity of Fourier transformation to numerically perturbed Type I collagen models (cyan) compared to the original one (black). Each time, the Cα atom coordinates of five residues are randomly disturbed by 5 Å affecting the deformation scores of ten steps compared to the original model. The major peak at 4 is preserved.
